# EZH2 overexpression is associated with aggressive behavior and promotes cell proliferation in CNS WHO grade 3 meningiomas

**DOI:** 10.1093/noajnl/vdaf112

**Published:** 2025-06-05

**Authors:** Péter Szőke, Dániel Sztankovics, Titanilla Dankó, Alzahra Ahmed Mohammed, Loránd Váncza, Gergő Papp, Ágnes Márk, Csaba Bödör, Anna Sebestyén, Katalin Dezső, Bálint Scheich

**Affiliations:** Department of Pathology and Experimental Cancer Research, Semmelweis University, Budapest, Hungary; Department of Pathology and Experimental Cancer Research, Semmelweis University, Budapest, Hungary; Department of Pathology and Experimental Cancer Research, Semmelweis University, Budapest, Hungary; Department of Pathology and Experimental Cancer Research, Semmelweis University, Budapest, Hungary; Department of Pathology and Experimental Cancer Research, Semmelweis University, Budapest, Hungary; Department of Pathology and Experimental Cancer Research, Semmelweis University, Budapest, Hungary; Department of Pathology and Experimental Cancer Research, Semmelweis University, Budapest, Hungary; HCEMM-SE Molecular Oncohematology Research Group, Department of Pathology and Experimental Cancer Research, Semmelweis University, Budapest, Hungary; Department of Pathology and Experimental Cancer Research, Semmelweis University, Budapest, Hungary; Department of Pathology and Experimental Cancer Research, Semmelweis University, Budapest, Hungary; Department of Pathology and Experimental Cancer Research, Semmelweis University, Budapest, Hungary; Department of Pathology and Experimental Cancer Research, Semmelweis University, Budapest, Hungary

**Keywords:** CNS WHO grade 3 meningioma, EPZ-6438, EZH2, H3 K27me3, IOMM-Lee

## Abstract

**Background:**

Central nervous system (CNS) World Health Organization (WHO) grade 3 meningiomas are aggressive tumors of the CNS whose clinical management is particularly challenging due to the lack of effective systemic treatment options. Recent multiomic studies revealed increasing expression and activity of the histone methyltransferase enhancer of zeste homolog 2 (EZH2) in parallel with the increase in meningioma grade. This study primarily aims to characterize in detail the clinical and molecular correlates of EZH2 expression in grade 3 meningiomas and to investigate its functional role both in vitro and in vivo.

**Methods:**

EZH2 expression was correlated with clinical outcome data as well as with the loss of p16 and H3 K27me3, 9p21.3 deletion, and *pTERT* mutational status in a cohort of 49 grade 3 meningiomas. Effects of the EZH2 methyltransferase inhibitor EPZ-6438 were investigated on the IOMM-Lee human grade 3 meningioma cell line.

**Results:**

Higher levels of EZH2 overexpression were associated with shorter overall and local progression-free survival and higher proliferative activity in our grade 3 meningioma cohort. Deletion of the 9p21.3 region and loss of H3 K27me3 were related to higher EZH2 expression, while loss of p16 and *pTERT* mutations did not show significant correlation. EPZ-6438 treatment exerted concentration-dependent inhibitory effect on IOMM-Lee cell proliferation through inhibition of cell cycle progression, increased p21, and decreased FOXM1 expression. Upon *per os* EPZ-6438 treatment, a slight inhibition of tumor growth with different underlying mechanisms was detected in a mouse heterotopic xenograft model.

**Conclusions:**

Our findings demonstrate that EZH2 is functionally relevant in grade 3 meningiomas, primarily through promoting cell proliferation.

**Key Points**

- Increased EZH2 expression is associated with poor prognosis in central nervous system (CNS) World Health Organization (WHO) grade 3 meningiomas.
- The EZH2 inhibitor EPZ-6438 reduces IOMM-Lee tumor cell growth both in vitro and in vivo.
- EZH2 primarily promotes cell proliferation in CNS WHO grade 3 meningioma.

**Importance of the Study**

Anaplastic meningiomas (central nervous system [CNS] World Health Organization [WHO] grade 3) are the most aggressive tumors of the meninges causing significant morbidity and mortality. Despite current advances in surgical treatment and radiotherapy, effective systemic therapeutic options for CNS WHO grade 3 meningiomas remain lacking. Therefore, intense research efforts have been directed at the identification of novel therapeutic targets in these tumors. Besides genetic alterations, epigenetic dysregulation seems to be a fundamental component in the pathogenesis of high-grade meningiomas, and the pharmacological inhibition of these mechanisms might open new perspectives in their therapy. EZH2 is one of the best characterized epigenetic regulatory molecules of which inhibitors with multiple mechanisms of action are available, used in clinical practice, or investigated in ongoing trials. This is the first study providing detailed information regarding the functional relevance of EZH2 in CNS WHO grade 3 meningioma and will promote further research towards the effective pharmacotherapy of this devastating disease.

Meningiomas are the most common primary tumors of the central nervous system (CNS) in adults and are currently classified as grade 1–3 according to the World Health Organization (WHO) system (CNS WHO 5).^1^ The rarely occurring anaplastic meningiomas (CNS WHO grade 3) account for approximately 1%–3% of these tumors and are characterized by aggressive behavior, high recurrence rates, and significant mortality.^2^ However, treatment options are limited to surgical resection and radiotherapy. Several clinical trials are ongoing, but effective systemic treatment options remain lacking.^3^ Therefore, the identification of novel therapeutic targets is crucial.

CNS WHO grade 3 meningiomas can develop de novo or result from the transformation of lower-grade tumors. Data obtained from the comprehensive, multiomic studies of recent years allow a precise classification of meningiomas outperforming the classical prognostication based solely on histopathology.^4^ In addition, these investigations provided invaluable information regarding the biology of aggressive meningiomas and the mechanisms of meningioma progression. Based on the available knowledge, epigenetic dysregulation seems to be a fundamental component. Transcriptomic and DNA-methylation profiles suggestive of activation of the polycomb repressive complex 2 (PRC2), a central epigenetic regulator, have been shown to characterize de novo CNS WHO grade 2^5^ as well as particularly aggressive CNS WHO grade 3 meningiomas^6^ and to be associated with increasing meningioma grade.^7^

The major function of PRC2 is the methylation of histone H3 at the lysine 27 position, producing its trimethylated form (H3 K27me3), which is able to potently suppress the transcription of its target genes. The catalytic subunit of PRC2 is the histone methyltransferase enhancer of zeste homolog 2 (EZH2). Considering the aforementioned results, overactivation of EZH2 could be suggested in high-grade meningiomas, and an increase of EZH2 expression was indeed detected in parallel with increasing meningioma grade, both at the mRNA and protein levels.^5,8–10^

Increased activity of EZH2 is a common finding in various malignant tumors, resulting from overexpression, gain-of-function mutations, or inactivation of counteracting mechanisms.^11^ Among the available EZH2 inhibitors with multiple mechanisms of action, the cofactor-competitive inhibitor EPZ-6438 (Tazemetostat) has already entered clinical practice having been approved for the treatment of follicular lymphoma and epithelioid sarcoma.^12^ In follicular lymphoma, EZH2 overactivation is related to recurrent pathogenic mutations, while in epithelioid sarcoma, loss of components of the SWItch/sucrose nonfermentable (SWI/SNF) chromatin remodeling complex, an antagonist of PRC2, results in H3 K27me3 addiction in the tumor cells.^13^ Since EZH2 mutations are not characteristic in meningiomas, increased PRC2 activity is more likely to be related to EZH2 overexpression. It is also worth noting that inactivation of SWI/SNF complex occurs in a significant proportion of aggressive meningiomas.^6^

In this study, we aimed to investigate whether EZH2 expression levels correlate with clinical behavior and molecular features in a cohort of CNS WHO grade 3 meningiomas. In addition to being independent diagnostic criteria for CNS WHO grade 3 meningioma,^1^ homozygous deletions affecting *CDKN2A/B* genes (within the 9p21.3 chromosomal region) and mutations of the *TERT* gene promoter (*pTERT*) are associated with poor prognosis according to some recent data.^14–16^ Loss of H3 K27me3 immunopositivity has been linked to more aggressive behavior in lower-grade meningiomas, but its prognostic role in CNS WHO grade 3 meningioma is controversial.^16–19^ Our second major goal was to investigate the correlation of these molecular alterations with EZH2 expression as well as clinical outcome data in our cohort. Additionally, we explored the functional relevance of EZH2 using the human CNS WHO grade 3 meningioma cell line IOMM-Lee treated with EPZ-6438. IOMM-Lee has been widely used in meningioma research and is known to harbor *pTERT* hotspot mutation (c.-124C>T) and a 3.6-kb deletion affecting the *CDKN2A* gene (Chr9:21971209–21974828).^20^ We performed a complex analysis of the effects of EPZ-6438 on this cell line both in vitro and in vivo, using a heterotopic mouse xenograft model. Furthermore, we examined the signal transduction mechanisms underlying the actions of this compound in our models.

## Materials and Methods

### Tissue Samples and Clinical Data

The samples of 88 patients were included in this study, all of whom were operated in the National Institute of Mental Health, Neurology and Neurosurgery. 54 cases (period: 2018–2022) of CNS WHO grade 1–3 meningiomas were included in the “validation cohort,” the purpose of which was to reproduce the data known from the literature on our own samples (exclusively EZH2 H-score and Ki-67 index were analyzed in this cohort). All subsequent investigations were performed on the “CNS WHO grade 3 meningioma” cohort consisting of 49 cases (period: 2011–2022; 14 cases from the 2018–2022 period overlapped with the CNS WHO grade 3 group of the validation cohort). Formalin-fixed paraffin-embedded (FFPE) samples were retrieved from the archives of the Department of Pathology and Experimental Cancer Research of Semmelweis University. The hematoxylin and eosin (H&E) stained sections were reevaluated by 2 investigators (A.A.M. and B.S.) according to the current WHO criteria. Exclusively CNS WHO grade 3 meningiomas with a mitotic index of ≥20/10 high-power field (HPF) or 1.6 mm^2^ (equating ≥12.5/mm^2^) were included in the CNS WHO grade 3 meningioma cohort, and in the case of patients with multiple samples, the first samples meeting these criteria were selected. We did not apply any further selection, all cases from the described time period, that met the aforementioned criteria, were included. Clinical data including overall survival (OS), local progression-free survival (LPFS), gross total resection (GTR; defined as no residual tumor on postoperative imaging), de novo or transformed (ie, developed from a meningioma of lower grade) nature, localization, therapeutic interventions, and basic epidemiological features (age, gender) were retrieved from the electronic documentation of the patients. LPFS was defined as the time elapsed from the surgery until the first radiological (MRI or CT) detection of recurrence or until a significant increase (at least 25%) of residual tumor volume. OS data were available for all (49/49), while LPFS data for only 80% (39/49) of CNS WHO grade 3 meningioma cases, somewhat limiting the conclusions that can be drawn from our studies regarding LPFS.

### Immunohistochemistry

Immunohistochemistry (IHC) studies on patient samples, as well as FFPE samples from the mouse IOMM-Lee xenograft model (see below), were performed according to the standard laboratory practice on 2- to 3-μm-thick sections, using a Leica BOND-MAX automated immunostaining system (Leica Biosystems). Specifications of the used Aurora kinase B (AURKB), CD31, EZH2, FOXM1, H3 K27me3, Ki-67, p16, and p21 primary antibodies are detailed in Supplementary Table S1. Primary antibody binding was visualized using Leica BOND Polymer Refine Detection System (DS9800, Leica Biosystems).

IHC slides were digitalized using an automated PANNORAMIC 1000 digital slide scanner (3DHISTECH Ltd.). EZH2, Ki-67, AURKB, FOXM1, H3 K27me3, and p21 IHCs were subjected to quantitative evaluation using the NuclearQuant image analysis module of the QuantCenter image analysis platform (3DHISTECH Ltd.) operating under SlideViewer 2.7 advanced slide viewing software (3DHISTECH Ltd.). On patient samples, characterization of EZH2 immunopositivity was performed within 3 fields of 0.4 mm^2^, previously designated in hotspots showing the highest EZH2 positivity on each slide. Analyzed fields on the Ki-67 IHC slides were designated in approximately the same areas of the corresponding sample (usually, but not necessarily coinciding with Ki-67 hotspots). In case of xenograft slides, AURKB, CD31, FOXM1, H3 K27me3, Ki-67, and p21 positivity was analyzed within areas designated according to those previously used for mitotic index analyses (see below).

EZH2 expression in patient samples and FOXM1, H3 K27me3, and p21 expression in xenograft samples were expressed according to the H-score, calculated from the percentage of negative (0), weak (1+), moderate (2+), and strong (3+) positive nuclei according to the following equation:


$$\begin{array}{l} H-score=(3\cdot 3+\;\%\;)+(2\cdot 2+\;\%\;)+(1\cdot 1+\;\%\;) \end{array}$$

The mean of the values measured in the 3 designated areas of each sample was used for the analyses. In the case of CNS WHO grade 3 meningioma patient samples, tumors were classified as “EZH2-high” and “EZH2-low,” according to their H-score being higher or lower than the median value of the whole cohort. For analyses of Ki-67 and AURKB positivity, describing fractions of cells within certain cell cycle phases, the percentage of positive nuclei (positivity index) was used. To characterize vascular density in xenograft samples, the proportion of positive pixels in CD31 IHC slides was determined using the DensitoQuant module of the QuantCenter platform (3DHISTECH Ltd.).

H3 K27me3 and p16 IHCs in patient samples were evaluated visually by 2 trained investigators (K.D. and B.S.). The methodology for the evaluation of H3 K27me3 IHC is highly variable in the literature.^21^ In this study, samples containing at least 80% negative nuclei in at least 80% of the evaluable (ie, containing appropriate internal controls) areas of the whole slide were classified as “H3 K27me3 reduced” (Supplementary Figure S1A). p16 IHC was evaluated as “complete loss” if the tumor cells were completely negative in all evaluable areas (along with positivity of the internal controls), while as “partial loss” when large contiguous areas showed a loss in an otherwise positive tumor (Supplementary Figure S1B). Endothelial cells were used as internal positive controls during the evaluation of both IHC reactions.

### Fluorescent In Situ Hybridization

Fluorescent in situ hybridization (FISH) analyses were performed on 2- to 3-µm-thick sections of the FFPE samples using the ZytoLight SPEC CDKN2A/CEN 9 Dual Colour Probe (Z-2063, ZytoVision GmbH) covering a 315-kb segment within the 9p21.3 region, including the *CDKN2B* and *MTAP* genes besides *CDKN2A*. The previously described protocol^22^ included deparaffinization and citrate pretreatment of the sections, followed by digestion (37°C, 15 min, 10% pepsin). Denaturation was performed at 85°C for 10 min and then the hybridization at 37°C overnight. After washing steps and DAPI (Vector Laboratories Inc.) counterstaining, digital images were taken using a Nikon Eclipse E600 fluorescence microscope. Then, at least 100 nonoverlapping nuclei were evaluated (Supplementary Figure S1C) from all cases, and the proportions of the following categories were calculated (all other scenarios were excluded from the analysis):

- 2 9p21.3 and 2 CEN9 signals (including hyperploid cells without obvious proportion shift), considered as a normal ratio (“N”).
- 1 9p21.3 and ≥2 CEN9 signals, considered as hemizygous loss (“He”).
- 1 9p21.3 and 1 CEN9 signals, considered as monosomy (“M”).
- 0 9p21.3 and ≥2 CEN9 signals, considered as homozygous loss (“Ho”).

Then, a score was calculated using the following equation:


$$\begin{array}{l} 9p21.3\;\;\;score=1\cdot N+0.5\cdot He+0.5\cdot M+0\cdot Ho \end{array}$$

Samples with a score of 1–0.67 were classified as “retained 9p21.3.” Samples with a score between 0.67 and 0.33 were considered to carry hemizygous loss, while those with a score below 0.33 were considered to contain homozygous loss. Of note, in cases showing partial loss of p16, FISH was performed on the p16 negative area (using the subsequent native section after the p16 IHC slides). Consequently, some tumors harboring 9p21.3 deletions were undoubtedly spatially heterogeneous; however, given the putative significance of these subclones, we analyzed them together with tumors that were homogeneous in this respect.

### *pTERT* Mutational Analysis

For the Sanger sequencing of mutational hotspots (c.-124C>T and c.-146C>T) of the *pTERT* (Supplementary Figure S1D), the following custom-designed primers were used: forward 5′-CACCCGTCCTGCCCCTTCACCTT-3′ and reverse 5′-GGCTTCCCACGTGCGCAGCAGGA-3′. For details, see Supplementary Materials and Methods.

### In Vitro Treatments and Analyses of Cell Growth

IOMM-Lee human anaplastic meningioma cell line (CRL-3370, ATCC) was maintained using high-glucose Dulbecco’s modified Eagle medium (DMEM; Biosera), supplemented with 10% fetal bovine serum (FBS; Biosera), 2 mM L-glutamine (Biosera), and 100 UI/mL penicillin–streptomycin (Biosera) at 37°C in humidified air with 5% CO_2_. In order to validate the effects of EPZ-6438, in vitro tests were also performed on the BEN-MEN-1 human meningioma cell line (ACC 599, DSMZ), which was originally derived from a CNS WHO grade 1 meningioma,^23^ but its molecular features correspond to CNS WHO grade 3 as it was recently demonstrated.^24^ BEN-MEN-1 cells were maintained using low-glucose DMEM (Biosera) and 20% FBS.

In order to characterize the effect of EPZ-6438 on cell growth, Alamar blue (AB) and sulforhodamine B (SRB) tests were performed. In the former, the fluorescence of the metabolized AB is measured, correlating with the number of metabolically active cells. In the latter, the measured absorbance correlates with the total protein content. To perform these tests, cells were seeded into 96-well plates (10^3^ IOMM-Lee or 2 × 10^3^ BEN-MEN-1 cells/100 µL/well) with media containing 10 µM, 20 µM, or 40 µM EPZ-6438 (GC14062, GlpBio). 0.25% DMSO was used as a vehicle, which did not affect cell growth in any of the used tests (data not shown). The total treatment time was 120 h. Media were replaced with the corresponding fresh solutions at 72 h in each group. AB and SRB tests were performed as previously described.^25^ For details and for the method of live-cell imaging, see Supplementary Materials and Methods.

### Flow Cytometry Analyses

IOMM-Lee cells were seeded into T25 flasks and treated for 120 h with either 0.25% DMSO or 40 µM EPZ-6438 (with medium renewal at 72 h). Then, proportions of cells in the G0/G1, S, and G2/M phases of cell cycle as well as necrotic and apoptotic cell death (sub-G1) were assessed using propidium iodide (PI). Forward scatter (FSC) and side scatter (SSC) were also analyzed as previously described.^26^ For details, see Supplementary Materials and Methods.

### Fluorescent Immunocytochemistry and Cell Block Preparation

In order to perform Ki-67 fluorescent immunocytochemistry and cell blocks for H3 K27me3 immunocytochemistry, cells were treated with various concentrations of EPZ-6438 as described above. For details, see Supplementary Materials and Methods.

### Wes Simple Capillary Immunoassay

For protein extraction, IOMM-Lee cells were seeded into T25 flasks and treated for 120 h with 0.25% DMSO, 10 µM or 40 µM EPZ-6438 (with medium renewal at 72 h). Protein extraction and Wes Simple assay were performed as previously described,^26^ using primary antibodies labeling cyclinD1, EZH2, FOXM1, p21, p53, and β-actin (loading control) specified in Supplementary Table S2. For details, see Supplementary Materials and Methods.

### In Vivo Experiments

The heterotopic xenograft model was generated using 8- to 10-week-old male SCID mice (*n* = 56) bred in the Laboratory Animal House of the Department of Pathology and Experimental Cancer Research of Semmelweis University, kept in standard plastic cages at 24–25°C, under aseptic conditions and provided with rodent chow and water ad libitum. IOMM-Lee cells (5 × 10^5^ cells in 100 μL FBS-free DMEM) were subcutaneously injected into the right flank region. Treatment was started 9 days following the tumor cell injection and maintained for further 9 days (this treatment duration was designed to achieve optimal tumor size at the end of the experiment, without ulceration or any other complications). *Per os* administration was performed 2 times daily using a dose volume of 10 µL/g, with either EPZ-6438 (*n* = 26) of 500 mg/kg/day (250 mg/kg BID; B0084-462339, BOC Sciences) or vehicle (*n* = 30; 0.25% Na-carboxymethyl-cellulose and 0.1% Tween-80 in dH_2_O). The length and width of the tumors were measured using a digital caliper and tumor volume was calculated using the following equation:


$$\begin{array}{l} Tumor\;\;\;volume=\frac{Length\cdot Width^{2}}{2} \end{array}$$

At the end of treatment period, mice were euthanized with cervical dislocation, and following the measurement of tumor weights, FFPE blocks were produced. H&E sections from all samples were digitalized as previously described, and the mitotic index (mitoses/mm^2^) was assessed by manual counting on 3 randomly designated areas of 0.4 mm^2^. The percentage of necrosis was determined by the manual designation of necrotic and whole cross-section areas of the tumors. IHC analyses were performed on 10–10 representative cases selected from both groups using the methodology described above.

### Statistical Analyses

Before all statistical analyses, D’Agostino–Pearson test was used to test the normal distribution of data sets and Grubbs’ test to identify outliers (α = 0.05). Parametric datasets were analyzed using unpaired *t* test for the comparison of 2 groups and Brown–Forsythe ANOVA test followed by Dunnet’s T3 multiple comparison test for the comparison of more than 2 groups. In the case of nonparametric datasets, the Mann–Whitney test was used for the comparison of 2 groups. Survival analyses were performed using Kaplan–Meier curves and log-rank (Mantel–Cox) test as well as univariate and multivariate Cox proportional hazards regression models. The correlation between 2 datasets was analyzed using the Spearman method. Repeated-measures ANOVA with Geisser–Greenhouse correction and Tukey’s multiple comparisons test were used to analyze tumor volume changes in the in vivo experiment. Each presented experiment was evaluated from at least 3 independent measurements. *P* < .05 was considered to be the threshold of statistical significance in each test. All analyses were performed using GraphPad Prism v.10.3.1.

## Results

### Characteristics of EZH2 Expression in CNS WHO Grade 3 Meningiomas

Analyses in the validation cohort showed that EZH2 H-score is significantly higher in CNS WHO grade 2 meningiomas showing elevated (4–19/10 HPF) mitotic index compared to CNS WHO grade 1 tumors (*P* < .01), which is not true in CNS WHO grade 2 meningiomas with brain invasion, but without elevated mitotic activity (Supplementary Figure S2A and B). The highest degree of EZH2 immunopositivity was detected in the CNS WHO grade 3 group (Supplementary Figure S2B; *P* < .0001 vs. both CNS WHO grade 1 and 2). In accordance with these, significant correlation could be detected between the EZH2 H-score and the Ki-67 positivity index (Supplementary Figure S2C) when pooled data of the whole cohort were analyzed (**P* < *.0001).

**Figure 1.**
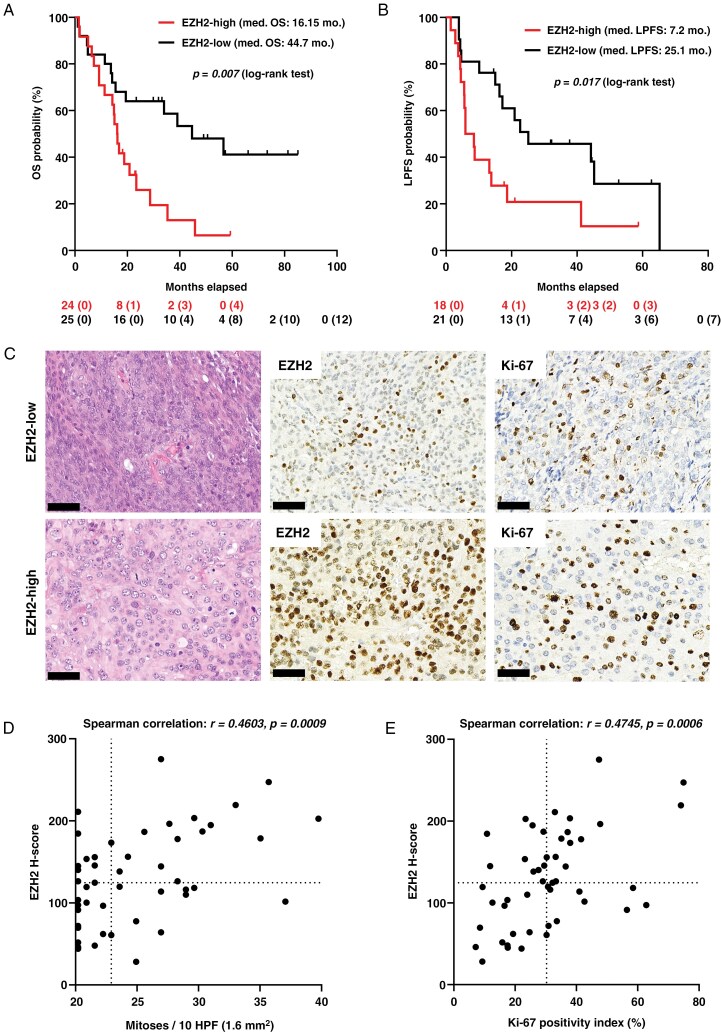
Kaplan–Meier curves representing the comparison of (A) overall survival (OS) and (B) local progression-free survival (LPFS) of EZH2-high and EZH2-low CNS WHO grade 3 meningioma cases [at the bottom: numbers at risk with the numbers of censored cases in brackets, log-rank (Mantel–Cox) test, med. = median, mo. = months]. (C) Representative images showing the morphology, EZH2, and Ki-67 immunopositivity of 1 EZH2-low and 1 EZH2-high case (scale bars: 50 μm). Scatter plots demonstrate the positive association of EZH2 *H*-score with (D) mitotic index and (E) Ki-67 positivity, both being confirmed by Spearman correlation (dashed lines show the median values). CNS, central nervous system; EZH2, enhancer of zeste homolog 2; WHO, World Health Organization.

Within the CNS WHO grade 3 meningioma cohort, the OS (Figure 1A; *P* < .01) and LPFS (Figure 1B; *P* < .05) of patients with EZH2-high tumors were significantly shorter compared to the EZH2-low group. Regarding major clinical and histopathological features, no apparent difference was seen, except for the considerably higher median mitotic index of EZH2-high tumors (Table 1). The positive correlation between EZH2 expression and mitotic as well as Ki-67 indices was already suggested by the pathological assessment of samples (Table 1, Figure 1C) and was then confirmed by the correlation analyses (Figure 1D; *P* < .001 and Figure 1E; *P* < .001, respectively). Among other clinical parameters, neither de novo, nor GTR status affected OS (Supplementary Figure S3A and C; *P* = .1747 and .3426, respectively), but both of these were associated with significantly longer LPFS (Supplementary Figure S3B and D; *P* < .05 regarding both). In the case of tumors showing high mitotic index (ie, higher than the median of the whole group), both OS (Supplementary Figure S3E; *P* < .001) and LPFS (Supplementary Figure S3F; *P* < .05) were significantly shorter. Univariate Cox regression analyses provided essentially the same results (Supplementary Tables S3 and S4). However, in multivariate models, also involving the molecular features (see below), only mitotic index was proven to be a significant predictor and only regarding OS, probably due to the low sample size and consequent low number of events per variable.

**Table 1.** Clinical, demographic, and histopathological characteristics of the CNS WHO grade 3 meningioma cohort

	EZH2-high	EZH2-low
Total number of cases	24 (100%)	25 (100%)
Gender		
Female	14 (58.3%)	13 (52%)
Male	10 (41.7%)	12 (48%)
Age (years)—median (range)	67 (14–83)	66 (43–86)
Recurrent tumor		
Yes (transformed)	11 (45.8%)	9 (36%)
No (de novo)	13 (54.2%)	16 (64%)
Localization		
Convexity	22 (91.7%)	18 (72%)
Falx	0	3 (12%)
Skull base	2 (8.3%)	1 (4%)
Tentorium	0	1 (4%)
Posterior fossa	0	2 (8%)
Multiple^a^		
Yes	5 (20.8%)	5 (20%)
No	19 (79.2%)	20 (80%)
Gross total resection (GTR)		
Yes	15 (62.5%)	17 (68%)
No	9 (37.5%)	8 (32%)
Therapy		
Radiotherapy	20 (83.3%)	18 (72%)
No oncotherapy	4 (16.7%)	7 (28%)
Mitoses/10 HPF (1.6 mm^2^)—median (range)	26.5 (20–40)	22 (20–37)
Necrosis		
Present	21 (87.5%)	21 (84%)
Absent	3 (12.5%)	4 (16%)
Brain invasion		
Present	9 (37.5%)	12 (48%)
Absent	15 (62.5%)	13 (52%)

CNS, central nervous system; EZH2, enhancer of zeste homolog 2; HPF, high-power field; WHO, World Health Organization.

^a^Multiple meningiomas were defined as 2 or more, spatially separated synchronous tumors.

### Correlations of EZH2 Expression and Molecular Features of CNS WHO Grade 3 Meningiomas

**Figure 2.**
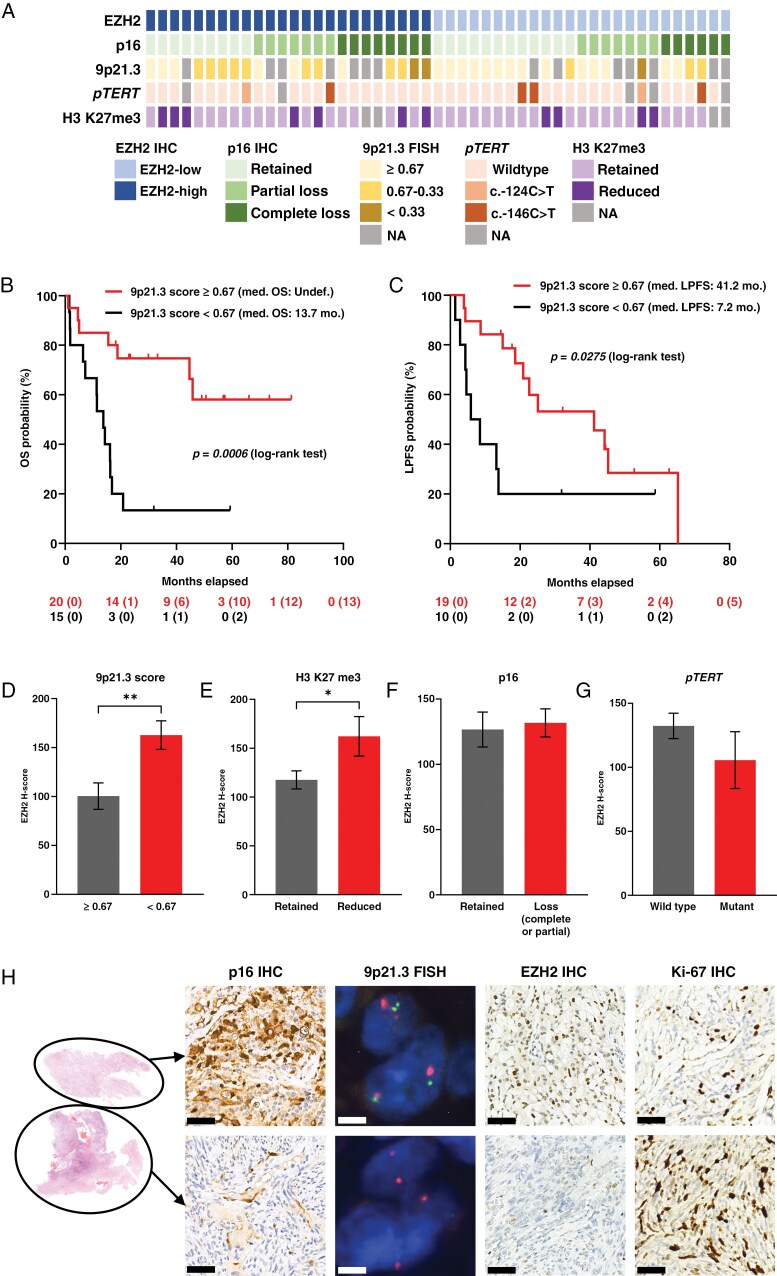
(A) Summary of molecular alterations in the CNS WHO grade 3 meningioma cohort (*n* = 49) clustered according to the EZH2-status (NA = not assessed). Kaplan–Meier curves representing the comparison of (B) overall survival (OS) and (C) local progression-free survival (LPFS) of 9p21.3 score ≥ 0.67 and <0.67 cases [at the bottom: numbers at risk with the numbers of censored cases in brackets, log-rank (Mantel–Cox) test, med. = median, mo. = months]. Analyses of EZH2 H-scores according to (D) 9p21.3 score [*n* = 20–15/group, means ± standard errors of the means (SEM); unpaired *t* test, **P < *.01*], (E) H3 K27me3 immunopositivity (*n* = 34–11/group, means ± SEM; unpaired *t* test, *P < *.05*), (F) p16 immunopositivity (*n* = 21–28/group, means ± SEM; Mann–Whitney test) and (G) *pTERT* mutational status (*n* = 38–36/group, means ± SEM; unpaired *t* test). (H) In 1 interesting case, partial p16 loss was detected (scale bars: 50 μm). Homozygous 9p21.3 deletion (green signal: 9p21.3, red signal: CEN9; scale bars: 5 μm) as well as lower EZH2 expression (scale bars: 50 μm) and higher Ki-67-index (scale bars: 50 μm) could be observed in the p16 negative (bottom row) compared to the p16 positive (upper row) area. CNS, central nervous system; EZH2, enhancer of zeste homolog 2; WHO, World Health Organization.

Molecular analyses performed in the CNS WHO grade 3 meningioma cohort revealed that the examined alterations overall occur with a higher frequency in EZH2-high tumors (Figure 2A; considering only those cases in which all molecular tests could be performed, the proportions of tumors without any alteration were 1/17 and 7/18 in EZH2-high and EZH2-low groups, respectively; *P* = .0408, Fisher’s exact test). During the analysis of the prognostic impact of the examined alterations, we combined the groups showing partial and complete p16 loss as well as 9p21.3 scores of 0.67–0.33 and ≤0.33, since no significant differences were seen between their behaviors (data not shown). According to survival analyses, the 9p21.3 status harbored the strongest prognostic value, since cases with 9p21.3 score < 0.67 showed significantly shorter OS (Figure 2B; *P* < .001) and LPFS (Figure 2C; *P* < .05). Interestingly, cases with p16 loss showed only a trend toward poorer prognosis (Supplementary Figure S4A and B; OS *P* = .1014; LPFS *P* = .1582). Behavior of *pTERT* mutant cases did not show significant difference compared to the wild-type group (Supplementary Figure S4C and D; OS *P* = .9946; LPFS *P* = .9086). Only slight trends toward shorter OS (Supplementary Figure S4E; *P* = .1747) and LPFS (Supplementary Figure S4F; *P* = .1060) were seen in the case of tumors showing reduced H3 K27me3 immunopositivity. Similar results were obtained using univariate Cox regression models, except that reduced H3 K27me3 immunopositivity proved to be a significant predictor regarding LPFS (Supplementary Tables S3 and S4).

Further analyses revealed significantly higher EZH2 H-scores in the 9p21.3 score < 0.67 (Figure 2D; *P* < .01) and H3 K27me3 reduced (Figure 2E; *P* < .05) groups. In contrast, p16 loss (Figure 2F; *P* = .7261) and *pTERT* mutations (Figure 2G; *P* = .3188) did not affect EZH2 expression. In a particularly interesting case (Figure 2H), heterogeneous p16 loss was detected using IHC, and FISH revealed a normal 9p21.3/CEN9 ratio (9p21.3 score = 0.68) in the p16 positive, while homozygous loss (9p21.3 score = 0.07) in the p16 negative area. Interestingly, EZH2 expression was remarkably higher within the area of intact p16 and 9p21.3, while the Ki-67-index was higher in the area with p16 and 9p21.3 losses, suggesting that these features can be highly heterogeneous and do not necessarily correlate within the same tumor as expected.

### Inhibitory Effect of EPZ-6438 on IOMM-Lee Tumor Cell Growth

**Figure 3.**
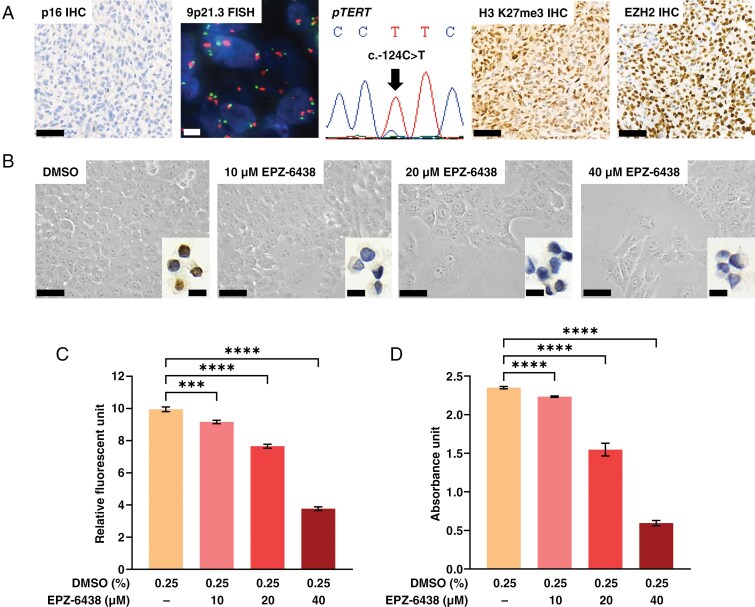
(A) Immunohistochemical and molecular analyses of an IOMM-Lee xenograft tumor sample revealed loss of p16 (scale bar: 50 μm), normal 9p21.3/CEN9 ratio (green signal: 9p21.3, red signal: CEN9; scale bar: 5 μm), *pTERT* c.-124C>T mutation, retained H3 K27me3 immunopositivity (scale bar: 50 μm) and exceedingly high EZH2 expression (scale bar: 50 μm). (B) Representative microscopic images showing decreasing confluence and appearance of enlarged IOMM-Lee cells in response to increasing concentrations of EPZ-6438 at the end of the 120-h treatment (scale bars: 25 μm). Inserts show representative cell groups demonstrating loss of H3 K27me3 immunopositivity at all used concentrations (scale bars: 10 μm). (C) Fluorescence in the Alamar blue (AB) test (correlating with the number of metabolically active cells) and (D) absorbance in the sulforhodamine B (SRB) test (correlating with total protein content) decreased in response to EPZ-6438 in a concentration-dependent manner (*n* = 11–12/group, means ± SEM; Brown–Forsythe ANOVA, Dunnet’s T3 multiple comparison test, ***P < *.001*, ****P < *.0001*). EZH2, enhancer of zeste homolog 2

Before starting in vitro experiments, we explored the molecular features of the IOMM-Lee cell line from tumor tissue obtained from the mouse xenograft model (Figure 3A). In accordance with the previously reported small *CDKN2A* deletion, tumor cells were completely negative with p16 IHC. 9p21.3 FISH showed multiple signals in the majority of tumor cells without obvious shift of the 9p21.3/CEN9 ratio (9p21.3 score = 0.91), confirming that the deletion is restricted to a small segment of the *CDKN2A* gene. *pTERT* sequencing confirmed the presence of c.-124C>T mutation. H3 K27me3 immunoreactivity was retained, and an extremely strong EZH2 immunopositivity could be detected.

In the in vitro experiments, EPZ-6438 exerted a concentration-dependent inhibitory effect on IOMM-Lee cell growth accompanied by the appearance of enlarged cells as readily detectable with the microscopic examination of cell cultures (Figure 3B). Similarly, live-cell imaging performed on 0.25% DMSO- (Supplementary File 2) and 40 μM EPZ-6438-treated cells (Supplementary File 3) showed the slower proliferation and enlargement of EPZ-6438-treated cells without obvious signs of cell death. Loss of H3 K27me3 immunopositivity was detected in the cell blocks at all used concentrations of EPZ-6438 (Figure 3B; *H*-score of nuclear positivity were 101.21 in DMSO, 1.95 in 10 µM, 6.87 in 20 µM, and 0.31 in 40 µM groups, respectively). In the AB test performed at 120 h of EPZ-6438 treatment (Figure 3C), a slight (7.9%) decrease in fluorescence (correlating with the number of metabolically active cells) was detected at 10 µM (*P* < *.001*) concentration, which reached 62.1% at 40 µM (*P* < *.0001*). A similar concentration-dependent decrease of absorbance (correlating with total protein content) was seen in the SRB test (Figure 3D), from 4.95% at 10 µM (*P* < *.0001*) to 74.7% at 40 µM (*P* < *.0001*). In the BEN-MEN-1 cell line, similar, but somewhat weaker effects of EPZ-6438 could be detected [at 40 µM, 35.5% decrease of fluorescence (*P* < *.0001*) and 44.7% decrease of absorbance (*P* < *.0001*) in the AB and SRB tests, respectively; Supplementary Figure S5A and B].

**Figure 4.**
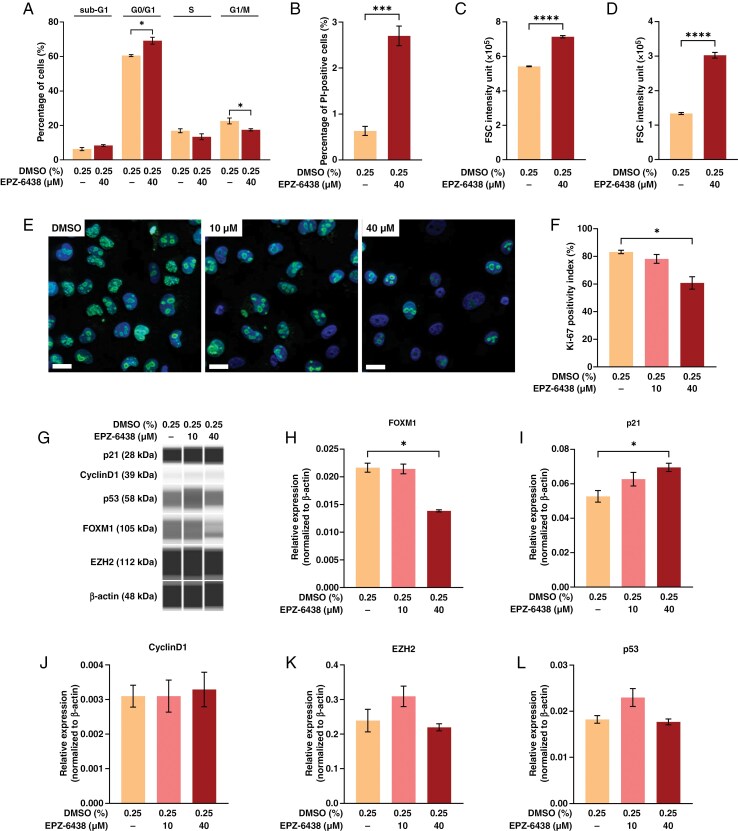
Flow cytometry analyses revealed (A) increased percentage of cells in the G0/G1 phases accompanied by decreased percentage of cells in G2/M phases upon 120-h treatment with 40 µM EPZ-6438. In addition, (B) increased percentage of PI-positive cells was detected during the measurement performed on unfixed cells, together with (C) increased forward scatter (FSC) and (D) side scatter (SSC) (*n* = 3/group, means ± SEM; unpaired *t* tests, *P < *.05*, ***P < *.001*, ****P < *.0001*). (E) Representative images showing a decrease of Ki-67 positivity index along with increasing concentration of EPZ-6438 (scale bars: 20 μm). (F) Quantitative analysis revealed that this effect was significant at 40 µM concentration (*n* = 4/group, means ± SEM; Brown–Forsythe ANOVA, Dunnet’s T3 multiple comparison test, *P < *.05*). (G) Virtual blot-like image demonstrating the results of 1 representative Wes Simple measurement on DMSO, 10 µM and 40 µM EPZ-6438-treated samples (left to right) by lane. Subsequent analyses revealed (H) decreased FOXM1 and (I) increased p21 relative expression at 40 µM EPZ-6438. In contrast, (J) cyclinD1, (K) EZH2, and (L) p53 expressions did not show significant change (*n* = 3/group, means ± SEM; Brown–Forsythe ANOVA, Dunnet’s T3 multiple comparison test, *P < *.05*). EZH2, enhancer of zeste homolog 2.

Flow cytometry revealed an increased proportion of cells in the G0/G1 phase (60.5% vs. 69.1%; *P* < *.05*) and a decreased percentage of cells in G2/M (22.6% vs. 17.5%; *P* < *.05*) in response to 120-h treatment with 40 µM EPZ-6438 (Figure 4A). The proportion of PI-positive cells when measured on unfixed cells was consistently higher upon 40 µM EPZ-6438 (*p* < *.001*), although the difference was numerically minimal (0.63% vs. 2.7%), querying the biological significance of necrotic-type cell death (Figure 4B). In addition, there was no difference regarding the proportion of cells in the sub-G1 population representing apoptotic cells (Figure 4A). The larger FSC (Figure 4C; *P* < *.0001*) and SSC (Figure 4D; *P* < *.0001*) upon EPZ-6438 treatment confirmed the increased cell size and granularity, respectively (representative measurements are demonstrated on Supplementary Figure S6A–D). The primary role of cell cycle inhibition in the effect of EPZ-6438 was also confirmed by the concentration-dependent decrease of Ki-67 positivity index (*P* < *.05* at 40 μM; Figure 4E and F), suggesting an increased number of cells in the G0 phase.

According to the results of Wes assay (Figure 4G), FOXM1 expression decreased (Figure 4H; *P* < *.05*) and p21 expression increased (Figure 4I; *P* < *.05*) in response to 120-h 40 µM EPZ-6438 treatment, while 10 µM did not exert significant effects. CyclinD1 (Figure 4J), EZH2 (Figure 4K), and p53 (Figure 4L) expression levels did not show significant changes (representative measurements are demonstrated in Supplementary Figure S7A–D).

### EPZ-6438 Inhibits IOMM-Lee Xenograft Tumor Growth In Vivo

**Figure 5.**
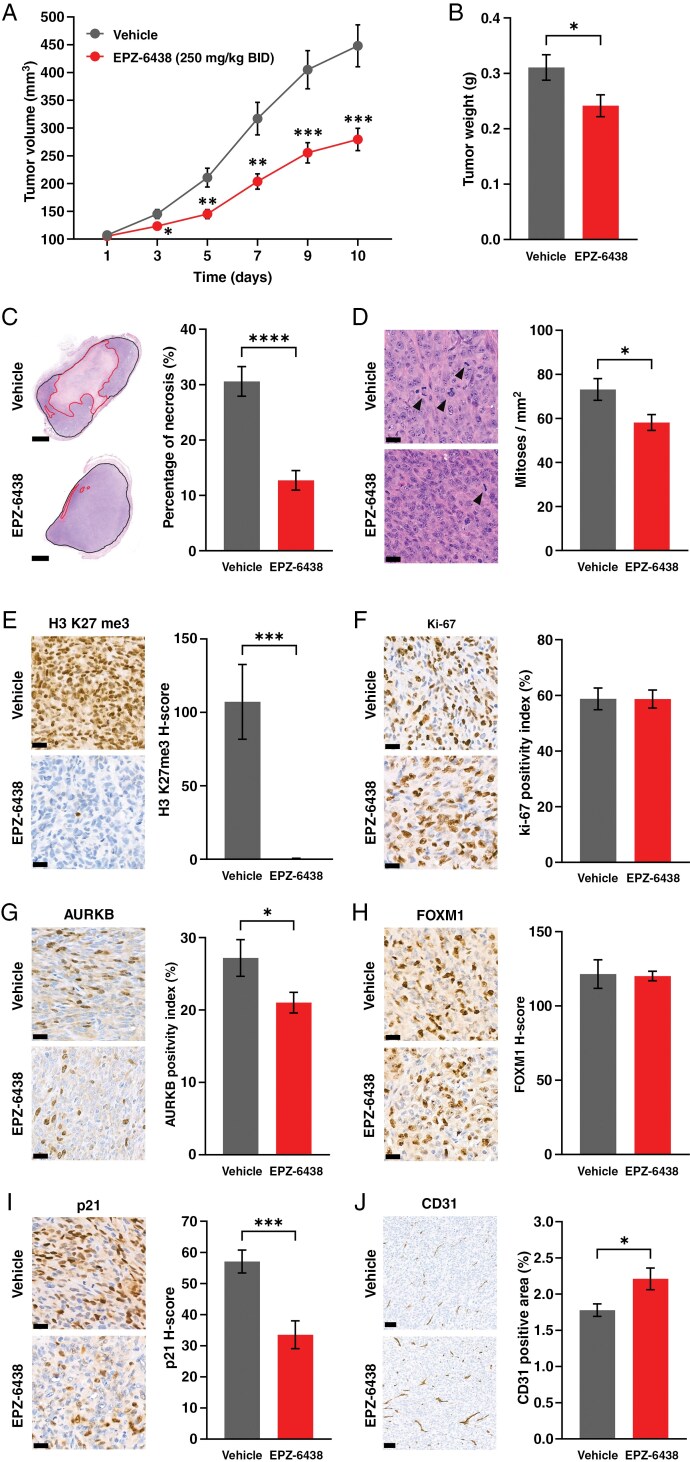
*Per os* EPZ-6438 treatment inhibited IOMM-Lee heterotopic xenograft tumor growth in SCID mice as it is demonstrated by (A) significantly smaller tumor volumes from the third day of treatment (*n* = 30–26/group, means ± SEM; 2-way repeated-measures ANOVA, Tukey’s multiple comparison test, *P < *.05*, **P < *.01*, ***P < *.001*) and (B) smaller tumor weights at the end of the experiment (*n* = 30–26/group, means ± SEM; Mann–Whitney test, *P < *.05*). (C) Proportion of necrotic areas was apparently higher in vehicle-treated tumors as it is demonstrated by representative images (scale bars: 1 mm) and results of morphometry (*n* = 30–26/group, means ± SEM; unpaired *t* test, ****P < *.0001*). (D) H&E morphology did not show significant difference (arrowheads point to mitotic figures, scale bars: 20 µm, also applicable to E–I), but mitotic index was lower in EPZ-6438-treated tumors (*n* = 30–26/group, means ± SEM; unpaired *t* test, *P < *.05*). (E) H3 K27me3 immunopositivity was virtually lost in the EPZ-6438-treated tumors (*n* = 10/group, means ± SEM; unpaired *t* test, ***P < *.001*). (F) There was no difference regarding Ki-67 positivity index (*n* = 10/group, means ± SEM; unpaired *t* test), but (G) the proportion of AURKB positive cells was lower in the EPZ-6438-treated tumors (*n* = 10/group, means ± SEM; unpaired *t* test, *P < *.05*). (H) FOXM1 expression was similar (*n* = 10/group, means ± SEM; unpaired *t* test), but (I) p21 immunopositivity was lower in EPZ-6438-treated compared to vehicle-treated tumors (*n* = 10/group, means ± SEM; unpaired *t* test, ***P < *.001*). (J) Tumor vascularity was slightly, but significantly higher in EPZ-6438-treated tumors (scale bars: 50 µm) as demonstrated by higher percentage of CD31 positive area (*n* = 10/group, means ± SEM; unpaired *t* test, *P < *.05*). H&E, hematoxylin and eosin.

In the IOMM-Lee mouse heterotopic xenograft model, tumor volumes were significantly smaller from the third day of treatment in the *per os* EPZ-6438-treated group (*P* < *.05*), and the growth inhibitory effect continued to increase over the next 6 days (Figure 5A; *P* < *.001* on the 10th day). At the end of the experiment, tumor weights were significantly smaller in the EPZ-6438-treated group (Figure 5B; *P* < *.05*).

Histologically, the most consistent difference was the strikingly more extensive necrosis in the vehicle-treated tumors, which was confirmed upon morphometry (Figure 5C; *P* < *.0001*). The mitotic activity of EPZ-6438-treated tumors was slightly, but significantly lower (Figure 5D; *P* < *.05*). Using IHC analyses, the effect of EPZ-6438 on the tumor cells could be validated since the H3 K27me3 immunoreactivity almost completely disappeared in response to the treatment (Figure 5E; *P* < *.001*). Interestingly, there was no difference regarding the Ki-67 positivity index (Figure 5F; *P* = *.9089*), although AURKB positivity was reduced in EPZ-6438-treated tumors (Figure 5G; *P* < *.05*). There was no difference in the FOXM1 immunopositivity (Figure 5H; *P* = *.9005*), and the p21 H-scores were significantly lower in the EPZ-6438-treated group (Figure 5I; *P* < *.001*). The percentage of CD31 positive area was slightly but significantly higher in EPZ-6438-treated tumors, suggesting increased tumor vascularity in this group (Figure 5J; *P* < *.05*).

## Discussion

The data presented here provide the first evidence that EZH2 overexpression is associated with an unfavorable outcome and higher proliferative activity as well as with 9p21.3 deletion and loss of H3 K27me3 in CNS WHO grade 3 meningiomas. The inhibition of IOMM-Lee tumor cell proliferation by EPZ-6438 suggests the functional relevance of EZH2 in CNS WHO grade 3 meningioma, although the mechanisms underlying the in vitro and in vivo effects seem to be different.

EZH2-high CNS WHO grade 3 meningiomas showed poorer OS and LPFS, although it must be acknowledged that the lack of an appropriate external validation cohort, which results from the study design, limits the generalizability of our data. Among the clinicopathological parameters examined, the most robust prognostic value was related to mitotic index, consistent with the existing literature.^16,18,27^ The significant correlations between the EZH2 expression, mitotic index, and Ki-67 index confirm that EZH2 overactivation contributes to tumor cell proliferation. These observations and the molecular correlates discussed below also suggest that EZH2 positivity is not an independent prognostic factor, but EZH2 IHC might be useful for identifying the anomalous minority of cases (ie, lower proliferation and high EZH2 expression or vice versa), and for the characterization of subclonal differences that might be clinically relevant, as demonstrated be the case showing heterogeneous p16 loss. Data regarding the prognostic impact of GTR as well as de novo or transformed nature in CNS WHO grade 3 meningiomas are controversial in the literature.^2,27–29^ It is worth noting, that these clinical features did not affect the OS, but GTR and de novo tumors showed longer LPFS in our cohort.

Hemi- and homozygous deletions affecting the 9p21.3 region were associated with a poor prognosis regarding both OS and LPFS. In contrast, tumors showing partial or complete loss of p16 only showed a trend toward unfavorable behavior. Loss of p16 has been shown to be a sensitive and specific marker of *CDKN2A* deletions, including microdeletions, in CNS WHO grade 3 meningiomas.^16,30^ Previous, usually array-based data consistently showed that homozygous *CDKN2A/B* deletions are accompanied by poor prognosis.^15,16^ In addition, recent data revealed that heterozygous deletions of these genes are associated with a similar prognosis to homozygous loss.^31^ In this study, we aimed to characterize separately the effects of p16 protein loss (the product *CDKN2A*), and larger chromosomal deletions detected by the 9p21.3 FISH probe covering a larger segment within the 9p21.3 region. Our observations emphasize the greater relevance of larger chromosomal lesions, suggesting that 1 or more adjacent genes, such as *CDKN2B* and *MTAP*, are at least as important as *CDKN2A*. Since we combined 9p21.3 score 0.67–0.33 and <0.33 cases during the survival analyses and the vast majority of them belonged to the former group, our data confirm the relevance of hemizygous deletions.

*pTERT* mutations did not significantly affect the survival data in our cohort and the value of reduced H3 K27me3 immunoreactivity could only be detected using univariate Cox regression and only regarding the LPFS. Within CNS WHO grade 3 meningiomas, these alterations have been shown to be associated with poor prognosis in previous studies, but conflicting findings can also be found in the literature.^16–19^ In the case of H3 K27me3 IHC, the methodology of evaluation (ie, whole slide or tissue microarray and the definition of loss) is quite diverse which could be the probable explanation of variable results.^21^ Regarding *pTERT* mutations, the low number of mutant tumors in our cohort limits the interpretability of this comparison.

Correlations between EZH2 expression and molecular features of the CNS WHO grade 3 meningioma cohort suggest a trend for a higher incidence of clinically relevant molecular alterations in EZH2-high tumors. Among these, hemi- and homozygous 9p21.3 deletions and loss of H3 K27me3 are related to higher EZH2 expression, while loss of p16 and *pTERT* mutations are not. These findings suggest that the loss of 9p21.3 genes other than *CDKN2A* is likely to be involved in the upregulation of EZH2. A possible player in this process is microRNA miR-31 encoded in the 9p21.3 region, which is frequently co-deleted with *CDKN2A* in melanoma, and inhibits EZH2 expression.^32^ To the best of our knowledge, the presented data are the first showing higher EZH2 expression in the context of H3 K27me3 loss. It is conceivable that this, even besides inhibited histone methyltransferase activity, alters the nonconventional, H3 K27me3-independent functions of the molecule (see below), although, the underlying mechanisms remain to be identified.

During in vitro experiments, EPZ-6438 reduced IOMM-Lee cell proliferation in a concentration-dependent manner, which was also detectable in the BEN-MEN-1 cell line. This effect was detected at higher concentrations compared to previously reported effective concentrations in other cell lines, such as diffuse large B-cell lymphoma,^33^ neuroblastoma,^34^ or rhabdoid tumor.^13^ In addition, H3 K27me3 immunopositivity was already lost at the lowest tested, barely effective concentration, suggesting that mechanisms besides inhibition of H3 methylation are also involved. A role for the inhibition of nonconventional EZH2 functions, such as methylation of nonhistone proteins or interactions with other regulatory molecules may be suggested,^35^ although the effect of EPZ-6438 on these is virtually unexplored.

Although not seen in our CNS WHO grade 3 meningioma cohort, the association of H3 K27me3 loss and poor prognosis in meningiomas of lower grades appears to contradict the inhibitory effect of EZH2 inhibition on IOMM-Lee tumor cell proliferation. Nevertheless, H3 K27me3 loss resulting from pharmacological EZH2 inhibition and intrinsic mechanisms can lead to opposite outcomes, as it was shown in H3 K27M-mutant diffuse midline glioma cell lines.^36^ However, other studies showed that EZH2 can also act as a tumor suppressor in this tumor, depending on the genetic context, and EPZ-6438 can even promote tumor cell proliferation.^37^ In conclusion, our results should be extrapolated with caution to meningiomas that, unlike the IOMM-Lee cells, show H3 K27me3 loss.

Further studies revealed that the in vitro effect is linked to inhibition of cell cycle progression accompanied by morphological changes, but without signs of significantly increased cell death. Protein expressional changes suggest that this is related to decreased FOXM1 and increased p21 expression. FOXM1 has recently been shown to be a central player in meningioma progression and proliferation by multiple lines of evidence,^38,39^ and its expression can be promoted by EZH2.^40^ p21 is a well-known inhibitor of cell cycle progression of which the major positive regulator is p53.^41^ However, p53 expression was not significantly affected by EPZ-6438, suggesting a p53-independent mechanism of p21 upregulation. This may involve the inhibition of EZH2-mediated repression of p21^42,43^ or an indirect pathway through FOXM1 also being able to decrease p21 protein levels in a p53-independent manner^44^ as it was also described in IOMM-Lee cells.^39^

The inhibitory effect of EPZ-6438 on tumor growth detected in the IOMM-Lee xenograft model was mediated by different mechanisms than those described in vitro. The in vivo effect was observed at a dose previously shown to be effective in rhabdoid tumor models.^13^ The tissue concentration of the compound was presumably lower than that having been effective in vitro, explaining the lack of effect on Ki-67 and FOXM1 positivity. However, the mitotic index and AURKB (expressed in G2 and M phases^45^) immunopositivity were lower in EPZ-6438-treated tumors, suggesting a longer inter-mitotic cycle time together with the unaltered Ki-67 index. The most striking difference was the smaller proportion of necrotic areas in EPZ-6438-treated tumors, for which a potential explanation is slower tumor growth. Another potential mechanism could be altered tumor vascularization, which is supported by increased CD31 positive area in EPZ-6438-treated tumors. EZH2 has been shown to promote tumor angiogenesis,^46^ while in other models, such as limb ischemia, opposite activity was described as well.^47^ The common ground in these was vascular normalization induced by EZH2 inhibition which has been shown to be able to inhibit tumor growth^48^ and might have also been an important mechanism in our model. In addition, EPZ-6438 treatment might have altered the metabolic or hypoxic adaptation of tumor cells to demands increasing with tumor growth.^49,50^ Lower p21 expression in EPZ-6438-treated tumors was another finding opposing in vitro results. However, this is likely to be rather related to poorer oxygen and nutrient supply of cells in vehicle-treated tumors, not directly to the effect of EPZ-6438. This observation might be important, because hypoxia-induced p21 upregulation has been shown to induce radioresistance.^51^

Our data provide the first solid and experimental evidence for the crucial importance of EZH2 in the malignant behavior of CNS WHO grade 3 meningiomas. EZH2 overexpression correlates with cell proliferation as well as 9p21.3 deletions and loss of H3 K27me3, although further mechanisms are undoubtedly involved. EPZ-6438 primarily inhibits cell cycle progression and proliferation in vitro in IOMM-Lee tumor cells, along with decreased FOXM1 and increased p21 expression. It must be emphasized, however, that this effect was seen at relatively high concentrations and did not induce significant cell death. Some features of the treated IOMM-Lee cells (enlargement and increased granularity, cell cycle arrest, increased p21 expression) are consistent with a quiescent or senescent phenotype, both of which are known to inhibit or promote tumor progression in a context-dependent manner.^52,53^ In addition, complete tumor regression could not be observed in response to the in vivo EPZ-6438 treatment either, although, some of the observed alterations in treated tumors could be clinically beneficial. These considerations suggest that cofactor-competitive inhibition of EZH2 alone may not be sufficient for the effective treatment of CNS WHO grade 3 meningiomas. In addition, other pathogenic mechanisms cooperating or competing with EZH2 cannot be neglected. Nevertheless, further investigation of nonconventional EZH2 functions and the effects of inhibitors with different mechanisms of action, as well as combination treatments, may open promising new perspectives in the future pharmacotherapy of this devastating disease.

## Supplementary Material

vdaf112_suppl_Supplementary_Tables_S1-S4_Figures_S1-S7_Files_2-3

## Data Availability

The data of this study will be made available upon reasonable request (Department of Pathology and Experimental Cancer Research, Semmelweis University, Üllői út 26, H-1085 Budapest, Hungary).
