# Supplementary material for: EZH2 overexpression is associated with aggressive behavior and promotes cell proliferation in CNS WHO grade 3 meningiomas
Source: Neurooncol Adv. 2025 Jun 5;7(1):vdaf112. doi: 10.1093/noajnl/vdaf112 (PMC12290454; doi:10.1093/noajnl/vdaf112)
Supplement: vdaf112_suppl_Supplementary_Tables_S1-S4_Figures_S1-S7_Files_2-3 [file vdaf112_suppl_supplementary_tables_s1-s4_figures_s1-s7_files_2-3.zip › NOA-D-25-00066R1_Supplementary File Legends.docx]

**Supplementary Material**

**Supplementary file 1**

Supplementary Materials and Methods, Supplementary Tables S1-4 and Supplementary Figures S1-7.

**Supplementary file 2**

Video obtained using live-cell imaging of IOMM-Lee cells treated with vehicle (0.25% DMSO) for 120 h (with medium renewal at 72 h).

**Supplementary file 3**

Video obtained using live-cell imaging of IOMM-Lee cells treated with 40 μM EPZ-6438 for 120 h (with medium renewal at 72 h).
