# Supplementary material for: EZH2 overexpression is associated with aggressive behavior and promotes cell proliferation in CNS WHO grade 3 meningiomas
Source: Neurooncol Adv. 2025 Jun 5;7(1):vdaf112. doi: 10.1093/noajnl/vdaf112 (PMC12290454; doi:10.1093/noajnl/vdaf112)
Supplement: vdaf112_suppl_Supplementary_Tables_S1-S4_Figures_S1-S7_Files_2-3 [file vdaf112_suppl_supplementary_tables_s1-s4_figures_s1-s7_files_2-3.zip › NOA-D-25-00066R1_Supplementary_file_1_clean.pdf]

Supplementary File 1

EZH2 overexpression is associated with aggressive behavior and promotes cell proliferation in CNS WHO grade 3 meningiomas

Péter Szőke, Dániel Sztankovics, Titanilla Dankó, Alzahra Ahmed Mohammed, Loránd Váncza, Gergő Papp, Ágnes Márk, Csaba Bödör, Anna Sebestyén, Katalin Dezső, Bálint Scheich*

Department of Pathology and Experimental Cancer Research, Semmelweis University, Üllői út 26., H-1085, Budapest, Hungary (P.Sz., D.Sz., T.D., A.A.M., L.V., G.P., Á.M., Cs.B., A.S., K.D., B.S.)

HCEMM-SE Molecular Oncohematology Research Group, Department of Pathology and Experimental Cancer Research, Semmelweis University, Üllői út 26., H-1085, Budapest, Hungary (Cs.B.)

**Corresponding author*

Supplementary Materials and Methods

pTERT mutational analysis

DNA was isolated from the FFPE samples using NucleoSpin DNA FFPE XS (740980, Macherey-Nagel). The region of interest was amplified in a Biometra thermal cycler. Following the purification of PCR products with Exo-SAP IT (78201, Thermo Fisher Scientific), sequencing was performed following NucleoSEQ purification (740523, Macherey-Nagel), using BigDye Terminator Cycle Sequencing kit (4337455, Thermo Fisher Scientific), on an ABI3500 platform (Thermo Fisher Scientific). Results were evaluated using the BioEdit Software.

Alamar blue (AB) and sulforhodamine B (SRB) tests

At the end of 120-h EPZ-6438 treatment, 10 µL/well AB solution (Thermo Fisher Scientific) was added, and fluorescence was measured following a 4-h incubation at 37°C using Fluoroskan Ascent FL fluorimeter and Ascent Software (Labsystems) in 570–590 nm wavelength range. For SRB assay, cells were fixed with 4°C 10% trichloroacetic acid for 60 min, washed with tap water and stained using 0.4% SRB for 15 min (Sigma-Aldrich, dissolved in 1% acetic acid, 50 µL/well). Following a washing step with 1% acetic acid, the protein-bound dye was redissolved in 10 mM Tris base solution (150 µL/well). Absorbance was measured using a Multiskan MS Microplate Reader and Transmit Software (Labsystems) at 570 nm wavelength.

Live-cell imaging

For live-cell imaging experiments, cells were seeded into T25 flasks (5 x 10^4 cells/flask) with either 0.25% DMSO or 40 µM EPZ-6438 and left to adhere for 4 h. Then, the flasks were placed on a CytoSMART^{TM} Lux2 inverted microscope, and bright field live-cell images were taken in 15-min intervals during the 116-h remaining treatment period (with medium renewal at 72 h). Finally, video files were generated with 0.1 s intervals between the images.

Flow cytometry analyses

Cells were harvested and treated with propidium iodide (PI, 1 mg/mL, Sigma-Aldrich) for 15 min in order to characterize necrotic-type cell death. In addition, forward scatter (FSC) and side scatter (SSC) were also measured to characterize cell size and granularity, respectively. The first measurement was followed by fixation of the cells in 70% ice-cold ethanol, alkalic extraction (200 mM Na_2HPO_4, pH 7.8), RNase treatment (100 µg/mL, Sigma-Aldrich) and

staining with PI (1 mg/mL) in order to analyze apoptotic-type cell death (sub-G1 fraction), as well as proportion of cells in the G0/G1, S and G2/M phases of cell cycle. Both measurements and analyses were performed using a Navios 8 color flow cytometer (Beckman Coulter) and Kaluza Software (Beckman Coulter). 2 x 10^4 events/measurement were acquired from all samples.

Fluorescent immunocytochemistry and cell block preparation

For fluorescent immunocytochemistry, IOMM-Lee cells were seeded into T25 flasks and treated with 0.25% DMSO, and 10 µM or 40 µM EPZ-6438 for 72 h. Then, cells were seeded into 6-well plates containing sterile 24 × 24 mm coverslips (5 x 10^4 cells/well), and appropriate treatments were continued for further 48 h. Following this, coverslips were fixed in 4% paraformaldehyde for 10 min at 4°C and treated with Triton-X (0.2%, 5 min, room temperature). Then, incubation with Ki-67 primary antibody (1:50, MIB-1, M7240, DAKO) for 1 h was followed by Alexa Fluor 488-labeled secondary antibody (1:200, anti-mouse polyclonal, A-21202, Invitrogen) for 30 min. Finally, coverslips were mounted using an aqueous fluorescence mounting medium (S3023, DAKO) and non-autofluorescent slides. All slides were scanned using a PANNORAMIC Confocal scanner (3DHISTECH Ltd.). On each digitalized slide (n=4/group), Ki-67 positivity index was determined using the CellQuant module of the QuantCenter platform (3DHISTECH Ltd.) with the analysis of at least 300 cells (2-5 fields of 1.6 mm^2/slide).

For cell block preparation, cells were harvested and 3 x 10^6 cells/group were fixed in 4% paraformaldehyde for 4 h, suspended in agar and embedded into paraffin. Then, H3 K27me3 IHC was performed and analyzed digitally on the whole cross-section area of each cell block to obtain H-scores, as previously described.

Wes™ Simple Capillary Immunoassay

Protein extraction from IOMM-Lee cells was performed with the lysis of cells (50 mM Tris, 10% glycerol, 150 mM NaCl; 1% Nonidet-P40, 10 mM NaF, 1 mM phenylmethylsulphonyl fluoride, 0.5 mM $NaVO_3$, pH=7.5) and protein concentrations were determined using the Bradford assay (BioRad). The 12–230-kDa Separation Module (ProteinSimple SM-W004) and either Anti-Rabbit (ProteinSimple DM-001) or Anti-Mouse Detection Kit (ProteinSimple DM-002) were used. Cell lysates of appropriate concentrations were diluted in Wes™ Sample Buffer

(ProteinSimple 042-195) and incubated with Fluorescent Master Mix (1:4, ProteinSimple PS-FL01-8) at 95°C for 5 min. The samples, the Antibody Diluent (ProteinSimple 042-203), the primary and secondary antibodies and the chemiluminescent substrate were pipetted into a Wes^{TM} capillary plate. Then, separation at 395 V for 30 min was followed by blocking for 5 min, incubation with primary antibodies and appropriate secondary antibodies for 30-30 min and chemiluminescence detection (luminol/peroxide) for 15 min. Compass Software was used to analyze the results, and electropherograms were manually corrected where needed.

Supplementary Table S1. Specifications of the primary antibodies used for the immunohistochemical studies on FFPE tissue samples.

Target name	Host species	Clone	Cat. No.	Source	Dilution
AURKB	Rabbit	N-term	1788-1	Epitomics	1:100
CD31	Rabbit	D8V9E	77699	Cell Signaling Technology	1:200
EZH2	Mouse	11/EZH2	612666	BD Biosciences	1:100
FOXM1	Rabbit	EPR17379	ab207298	Abcam	1:250
H3 K27me3	Rabbit	C36B11	9733	Cell Signaling Technology	1:400
Ki-67	Mouse	MIB-1	M7240	DAKO	1:100
p16	Mouse	E6H4	06695248001	Roche	RTU
p21	Rabbit	12D1	2947	Cell Signaling Technology	1:50

Supplementary Table S2. Specifications of the primary antibodies used for the Wes^{TM} Simple Capillary Immunoassay.

Target name	Host species	Clone	Cat. No.	Source	Dilution
β-actin	Mouse	AC-74	A2228	Sigma-Aldrich	1:50
CyclinD1	Rabbit	92G2	2978	Cell Signaling Technology	1:25
EZH2	Rabbit	D2C9	5246	Cell Signaling Technology	1:50
FOXM1	Rabbit	D12D5	5436	Cell Signaling Technology	1:50
p21	Rabbit	12D1	2947	Cell Signaling Technology	1:50
p53	Rabbit	Polyclonal	9282	Cell Signaling Technology	1:50

Supplementary Table S3. Univariate and multivariate Cox proportional hazards regression models estimating for overall survival (OS) in the CNS WHO grade 3 meningioma cohort. *p* values reaching the level of significance *(p<0.05)* were highlighted in bold. *(HR: Hazard ratio, CI: Confidence interval, GTR: Gross total resection.)*

**Predictor variables**	**N**	**OS**					
		**Univariate**			**Multivariate**		
		**HR**	**95% CI**	***p-value***	**HR**	**95% CI**	***p-value***
EZH2 H-score	49	1.009	1.003 - 1.015	**0.0039**	0.9994	0.9882 - 1.010	0.9155
Mitotic index	49	1.117	1.050 - 1.183	**0.0003**	1.135	1.001 - 1.284	**0.0431**
No GTR	49	1.399	0.6780 - 2.789	0.3469	0.2711	0.03576 - 1.666	0.1779
Transformed	49	1.603	0.7950 - 3.187	0.1785	2.578	0.5224 - 11.19	0.2169
9p21.3 score <0.67	35	4.672	1.843 - 12.89	**0.0016**	3.545	0.9445 - 16.56	0.0805
p16 lost	49	1.800	0.8969 - 3.784	0.1056	2.235	0.7002 - 7.712	0.1816
pTERT mutant	44	0.9952	0.2917 - 2.586	0.9929	1.357	0.1528 - 9.615	0.7629
H3 K27me3 reduced	45	1.720	0.7411 - 3.691	0.1798	1.691	0.3627 - 6.936	0.4782

Supplementary Table S4. Univariate and multivariate Cox proportional hazards regression models estimating for local progression-free survival (LPFS) in the CNS WHO grade 3 meningioma cohort. *p* values reaching the level of significance *(p<0.05)* were highlighted in bold. *(HR: Hazard ratio, CI: Confidence interval, GTR: Gross total resection.)*

**Predictor variables**	**N**	**LPFS**					
		**Univariate**			**Multivariate**		
		**HR**	**95% CI**	***p-value***	**HR**	**95% CI**	***p-value***
EZH2 H-score	39	1.007	1.001 - 1.013	**0.0321**	0.9931	0.9804 - 1.006	0.2830
Mitotic index	39	1.155	1.054 - 1.264	**0.0016**	1.161	0.9873 - 1.363	0.0634
No GTR	39	2.186	0.9809 - 4.676	**0.0471**	1.080	0.1067 - 17.54	0.9515
Transformed	39	2.364	1.109 - 5.037	**0.0243**	2.833	0.2042 - 22.41	0.3638
9p21.3 score <0.67	29	2.742	1.041 - 6.918	**0.0339**	4.685	1.013 - 26.03	0.0578
p16 lost	39	1.676	0.7916 - 3.648	0.1802	1.107	0.3812 - 3.202	0.8491
pTERT mutant	35	1.078	0.2539 - 3.145	0.9032	0.396	0.04471 - 2.610	0.3509
H3 K27me3 reduced	36	2.426	0.9589 - 5.723	**0.0485**	2.873	0.6029 - 11.95	0.1569

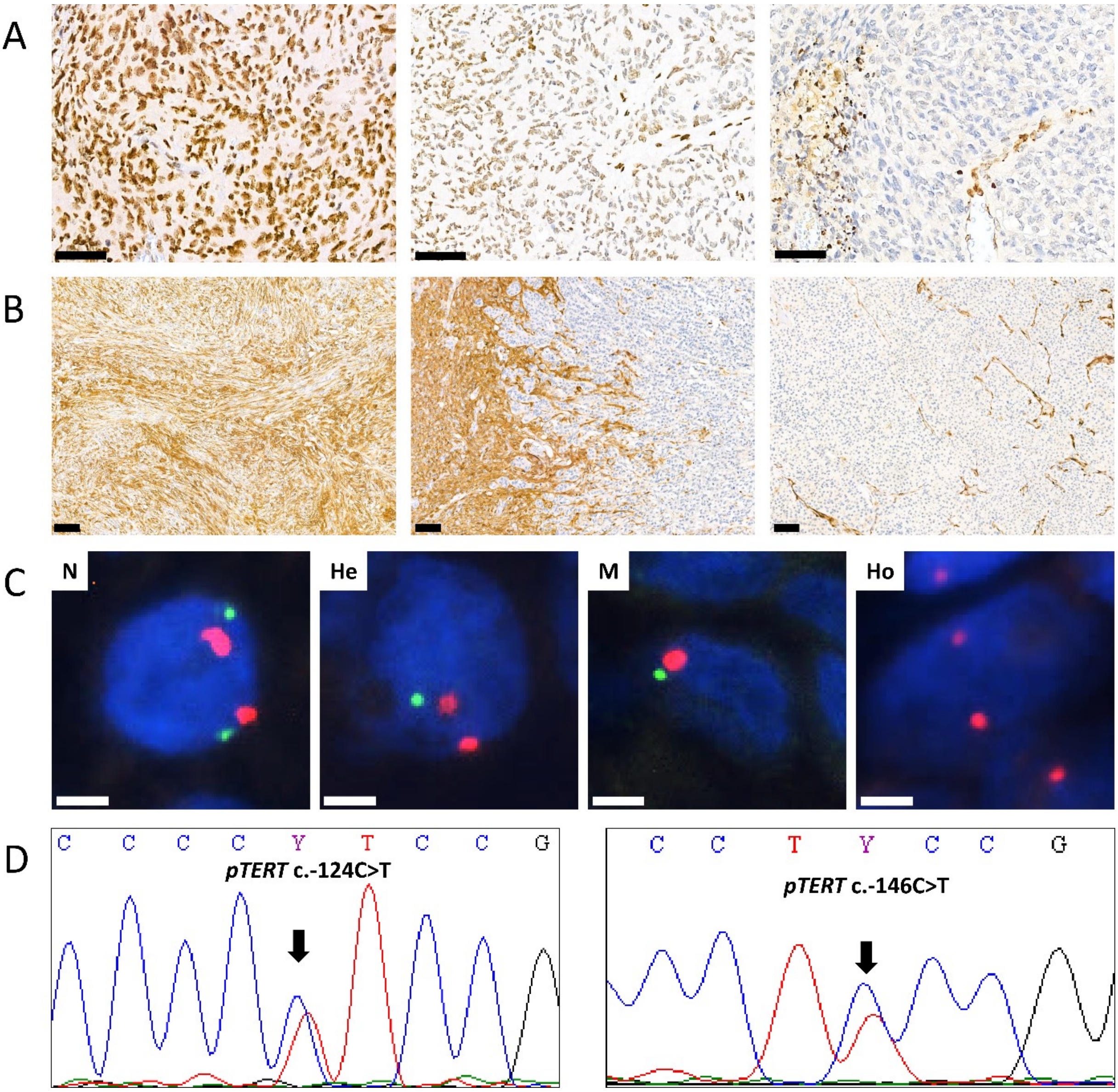

Supplementary Figure S1. Representative images demonstrate the methodology of H3 K27me3 and p16 immunohistochemistry as well as 9p21.3 FISH and *pTERT* mutational analysis. (**A**) Left panel shows a H3 K27me3 intact case with strong nuclear staining *(scale bars: 50 µm)*. Cases with weaker or uneven staining, like the one on the middle panel were also classified as H3 K27me3 retained. The right panel represents a H3 K27me3 reduced case with negativity of the tumor cells and positivity of the internal controls (endothelial cells). Staining of the perinecrotic nuclear debris (left side of the right panel) was disregarded. (**B**) Examples of retained p16 positivity (left panel; *scale bars: 100 µm*) as well as partial (middle panel) and complete p16 loss, with positivity of the internal controls (endothelial cells; right panel). (**C**) Examples of nuclei evaluated as normal 9p21.3/CEN9 ratio (N), hemizygous deletion (He),

monosomy (M) or homozygous deletion (Ho), respectively *(green signal: 9p21.3, red signal: CEN9; scale bars: 5 µm)*. (**D**) Examples of *pTERT* Sanger sequencing results in cases harboring c.-124C>T and c.-146C>T mutations, respectively.

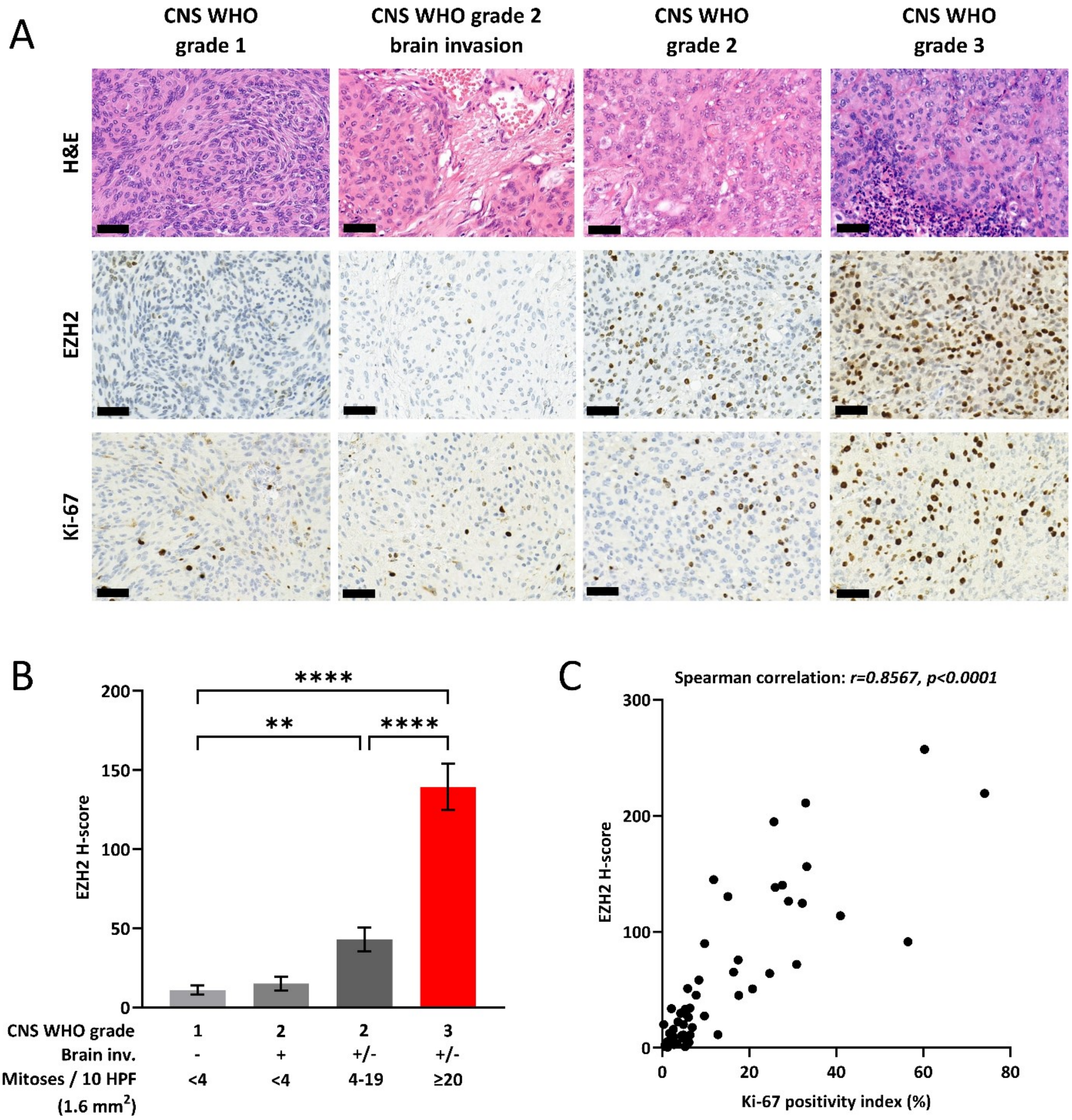

Supplementary Figure S2. Characteristics of EZH2 expression in the validation cohort. (**A**) Representative images showing the EZH2 and Ki-67 immunopositivity in CNS WHO grade 1-3 meningioma cases *(scale bars: 50 µm)*. (**B**) CNS WHO grade 2 meningiomas with elevated mitotic index showed higher EZH2 H-scores compared to CNS WHO grade 1 and grade 2 tumors with low mitotic index and brain invasion, respectively. The highest EZH2 H-scores (and highest variability) was detected in the CNS WHO grade 3 group *[n=12-16/group, means ± standard errors of the means (SEM); Brown-Forsythe ANOVA, Dunnet's T3 multiple comparison test, **p<0.01, ****p<0.0001]*. (**C**) Scatter plot demonstrate the positive association of EZH2 H-score with Ki-67 positivity index being confirmed by Spearman correlation.

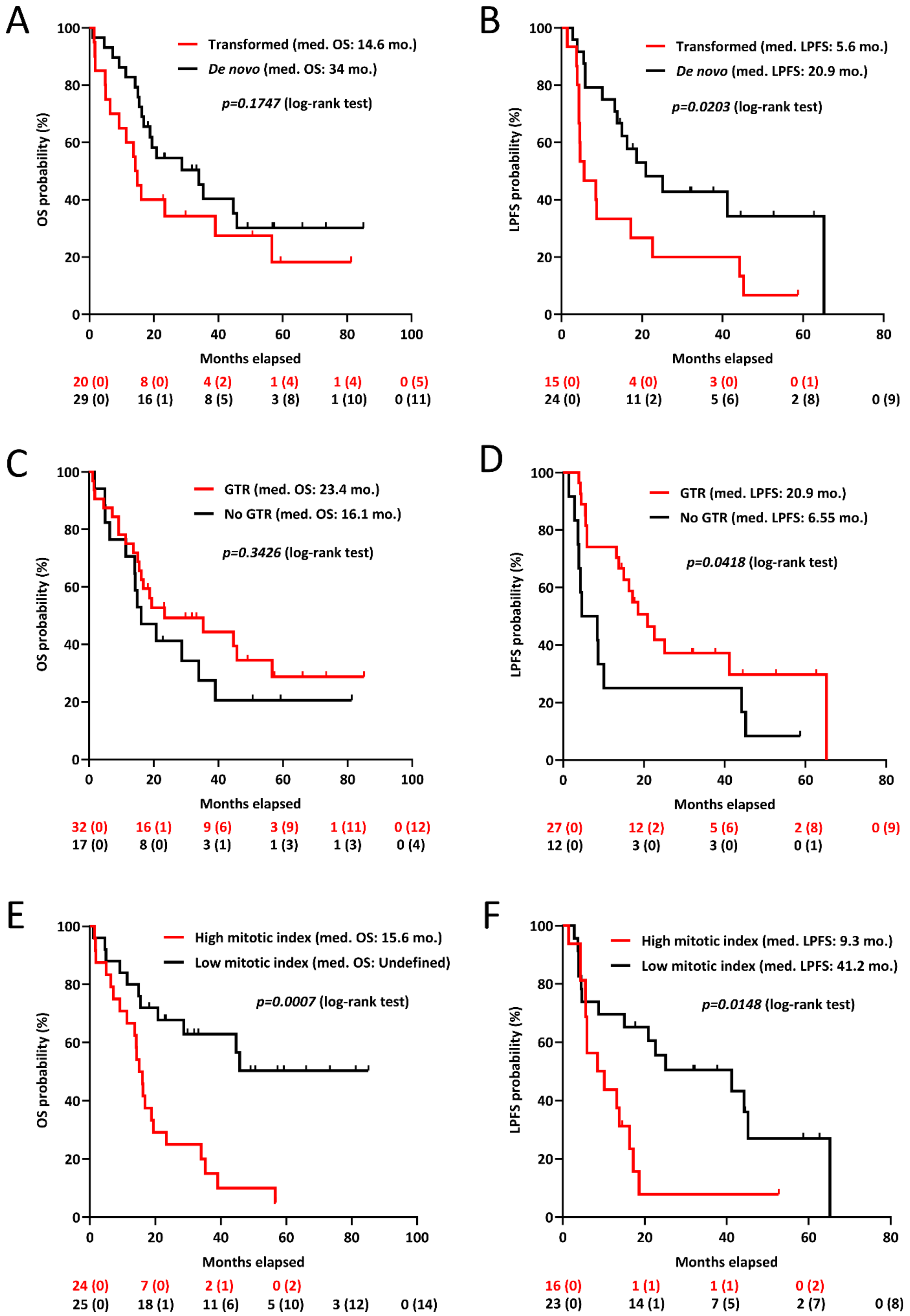
A
OS probability (%)
Months elapsed
Transformed (med. OS: 14.6 mo.)
De novo (med. OS: 34 mo.)
p=0.1747 (log-rank test)
20 (0) 8 (0) 4 (2) 1 (4) 1 (4) 0 (5)
29 (0) 16 (1) 8 (5) 3 (8) 1 (10) 0 (11)
B
LPFS probability (%)
Months elapsed
Transformed (med. LPFS: 5.6 mo.)
De novo (med. LPFS: 20.9 mo.)
p=0.0203 (log-rank test)
15 (0) 4 (0) 3 (0) 0 (1)
24 (0) 11 (2) 5 (6) 2 (8) 0 (9)
C
OS probability (%)
Months elapsed
GTR (med. OS: 23.4 mo.)
No GTR (med. OS: 16.1 mo.)
p=0.3426 (log-rank test)
32 (0) 16 (1) 9 (6) 3 (9) 1 (11) 0 (12)
17 (0) 8 (0) 3 (1) 1 (3) 1 (3) 0 (4)
D
LPFS probability (%)
Months elapsed
GTR (med. LPFS: 20.9 mo.)
No GTR (med. LPFS: 6.55 mo.)
p=0.0418 (log-rank test)
27 (0) 12 (2) 5 (6) 2 (8) 0 (9)
12 (0) 3 (0) 3 (0) 0 (1)
E
OS probability (%)
Months elapsed
High mitotic index (med. OS: 15.6 mo.)
Low mitotic index (med. OS: Undefined)
p=0.0007 (log-rank test)
24 (0) 7 (0) 2 (1) 0 (2)
25 (0) 18 (1) 11 (6) 5 (10) 3 (12) 0 (14)
F
LPFS probability (%)
Months elapsed
High mitotic index (med. LPFS: 9.3 mo.)
Low mitotic index (med. LPFS: 41.2 mo.)
p=0.0148 (log-rank test)
16 (0) 1 (1) 1 (1) 0 (2)
23 (0) 14 (1) 7 (5) 2 (7) 0 (8)

Supplementary Figure S3. Kaplan-Meier curves demonstrate that (**A**) overall survival (OS) is not significantly different, but (**B**) local progression-free survival (LPFS) is shorter in transformed CNS WHO grade 3 meningiomas compared to *de novo* cases. Similarly, (**C**) OS did not show significant difference but (**D**) LPFS was significantly shorter in no gross total resection (no GTR) *vs.* GTR cases. The most robust prognostic power was associated with mitotic activity; both (**E**) OS and (**F**) LPFS were significantly shorter in cases with high mitotic index (i.e. higher than the median value of the whole cohort) *[at the bottom: numbers at risk with the numbers of censored cases in brackets, log-rank (Mantel-Cox) test, med.=median, mo.=months]*.

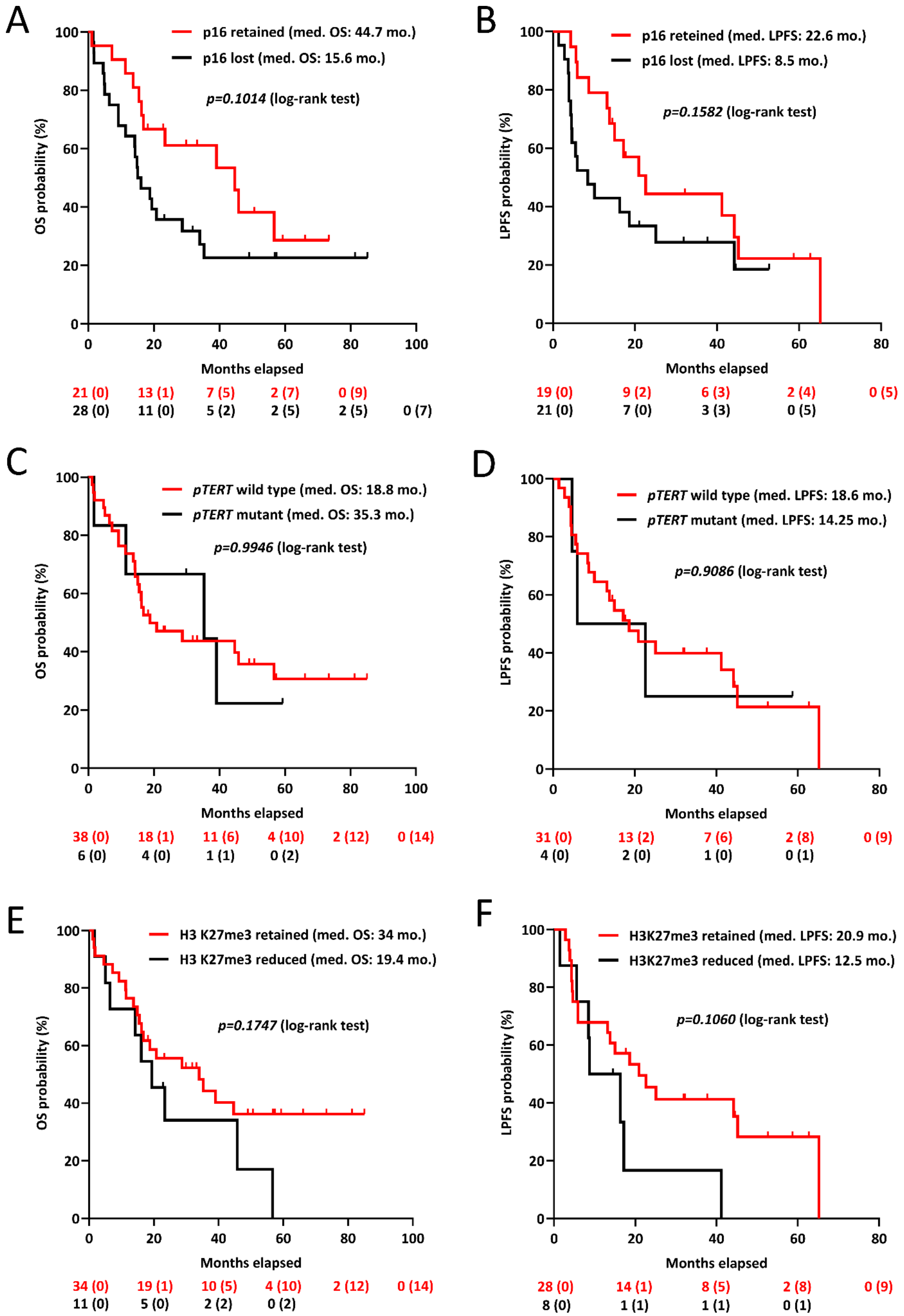
A
p16 retained (med. OS: 44.7 mo.)
p16 lost (med. OS: 15.6 mo.)
p=0.1014 (log-rank test)
OS probability (%)
Months elapsed
21 (0) 13 (1) 7 (5) 2 (7) 0 (9)
28 (0) 11 (0) 5 (2) 2 (5) 2 (5) 0 (7)
B
p16 reteined (med. LPFS: 22.6 mo.)
p16 lost (med. LPFS: 8.5 mo.)
p=0.1582 (log-rank test)
LPFS probability (%)
Months elapsed
19 (0) 9 (2) 6 (3) 2 (4) 0 (5)
21 (0) 7 (0) 3 (3) 0 (5)
C
pTERT wild type (med. OS: 18.8 mo.)
pTERT mutant (med. OS: 35.3 mo.)
p=0.9946 (log-rank test)
OS probability (%)
Months elapsed
38 (0) 18 (1) 11 (6) 4 (10) 2 (12) 0 (14)
6 (0) 4 (0) 1 (1) 0 (2)
D
pTERT wild type (med. LPFS: 18.6 mo.)
pTERT mutant (med. LPFS: 14.25 mo.)
p=0.9086 (log-rank test)
LPFS probability (%)
Months elapsed
31 (0) 13 (2) 7 (6) 2 (8) 0 (9)
4 (0) 2 (0) 1 (0) 0 (1)
E
H3 K27me3 retained (med. OS: 34 mo.)
H3 K27me3 reduced (med. OS: 19.4 mo.)
p=0.1747 (log-rank test)
OS probability (%)
Months elapsed
34 (0) 19 (1) 10 (5) 4 (10) 2 (12) 0 (14)
11 (0) 5 (0) 2 (2) 0 (2)
F
H3K27me3 retained (med. LPFS: 20.9 mo.)
H3K27me3 reduced (med. LPFS: 12.5 mo.)
p=0.1060 (log-rank test)
LPFS probability (%)
Months elapsed
28 (0) 14 (1) 8 (5) 2 (8) 0 (9)
8 (0) 1 (1) 1 (1) 0 (1)

Supplementary Figure S4. Kaplan-Meier curves demonstrate no significant difference in overall survival (OS) and local progression-free survival (LPFS) between cases showing [(**A**) and (**B**)] retained or lost (complete or partial) p16 immunopositivity, [(**C**) and (**D**)] carrying wild type or mutant *pTERT* and [(**E**) and (**F**)] showing retained or reduced H3 K27me3 immunopositivity *[at the bottom: numbers at risk with the numbers of censored cases in brackets, log-rank (Mantel-Cox) test, med.=median, mo.=months]*.

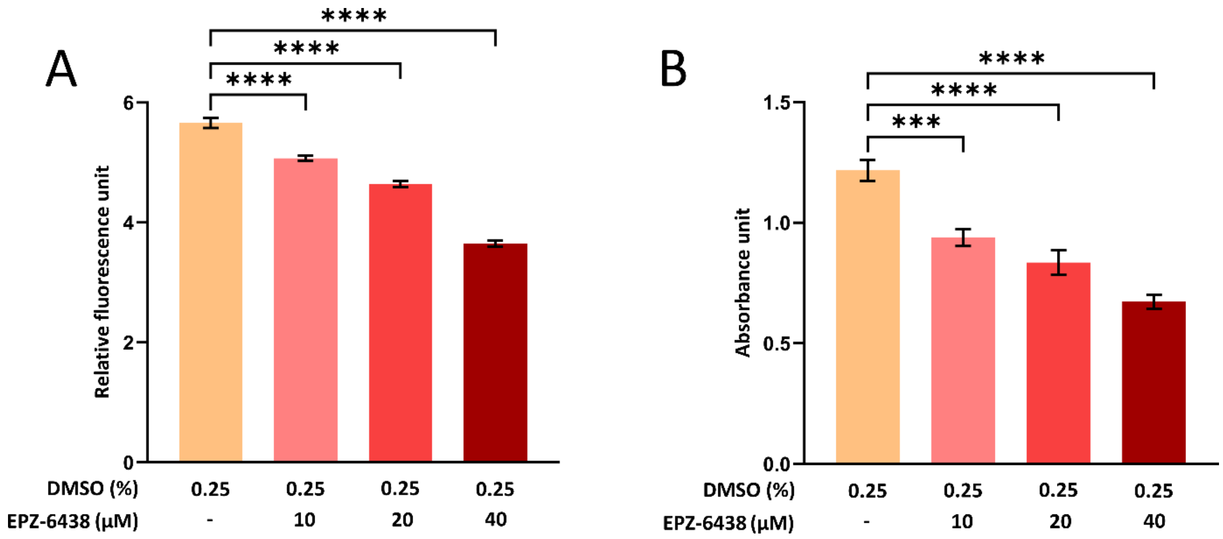

Supplementary Figure S5. Effects of EPZ-6438 on BEN-MEN-1 cell growth. (**A**) Fluorescence in the Alamar blue (AB) test (correlating with the number of metabolically active cells) and (**B**) absorbance in the sulforhodamine B (SRB) test (correlating with total protein content) decreased in response to EPZ-6438 in a concentration-dependent manner *(n=12/group, means ± SEM; Brown-Forsythe ANOVA, Dunnet's T3 multiple comparison test, ***p<0.001, ****p<0.0001).*

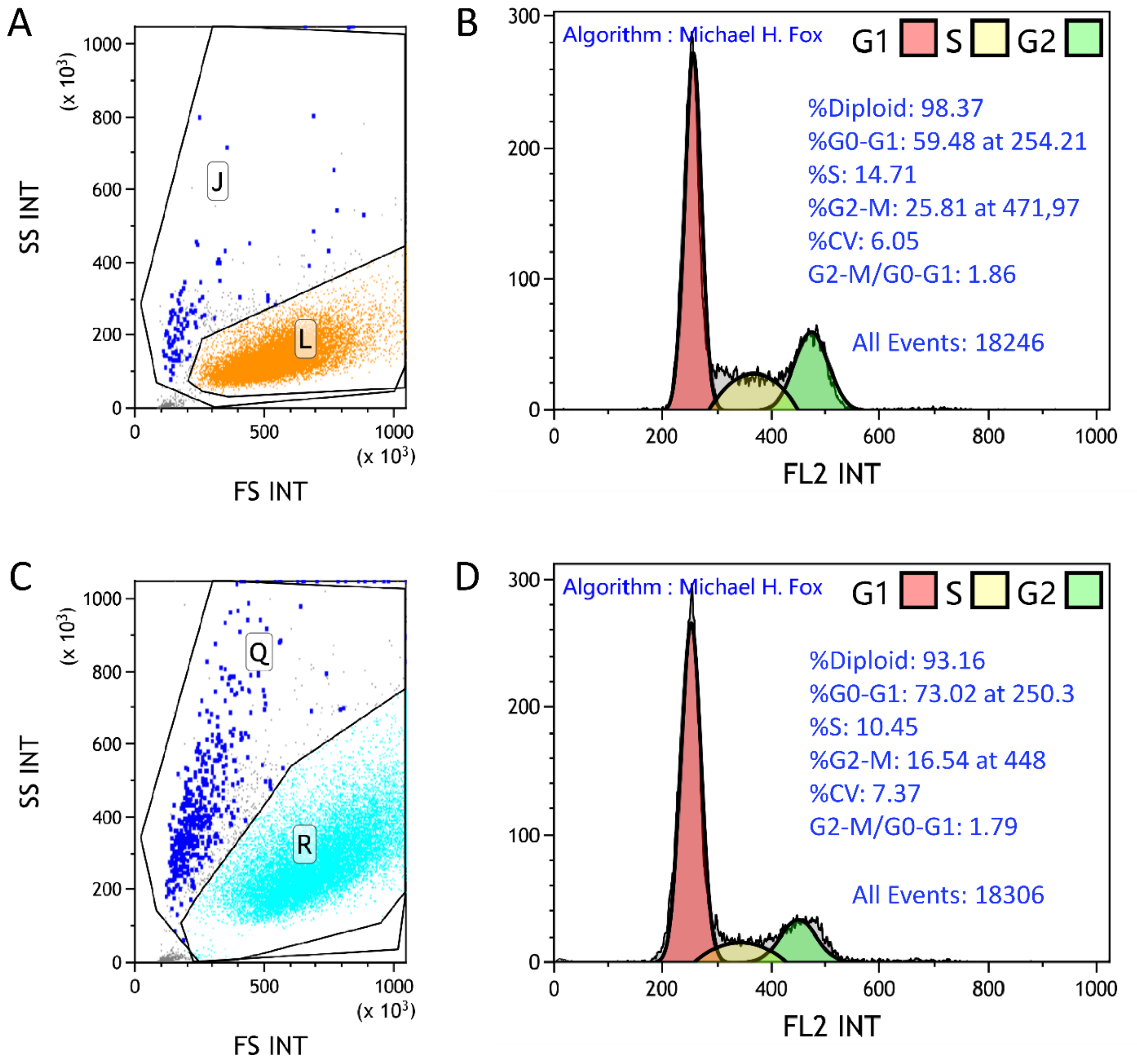

Supplementary Figure S6. Results of representative flow cytometry analyses. (**A**) Scatter plot demonstrates the side scatter intensity (SS INT) and forward scatter intensity (FS INT) of vehicle-treated (120 h) cells. Larger dark-blue dots represent unfixed propidium iodide (PI) positive cells. (**B**) Diagram demonstrating the distribution of cells within different phases of cell cycle in vehicle-treated (120 h) cells. (**C**) Scatter plot demonstrates the SS INT and FS INT of 40 µM EPZ-6438-treated (120 h) cells. Larger dark-blue dots represent unfixed PI positive cells. (**D**) Diagram demonstrating the distribution of cells within different phases of cell cycle in 40 µM EPZ-6438-treated (120 h) cells.

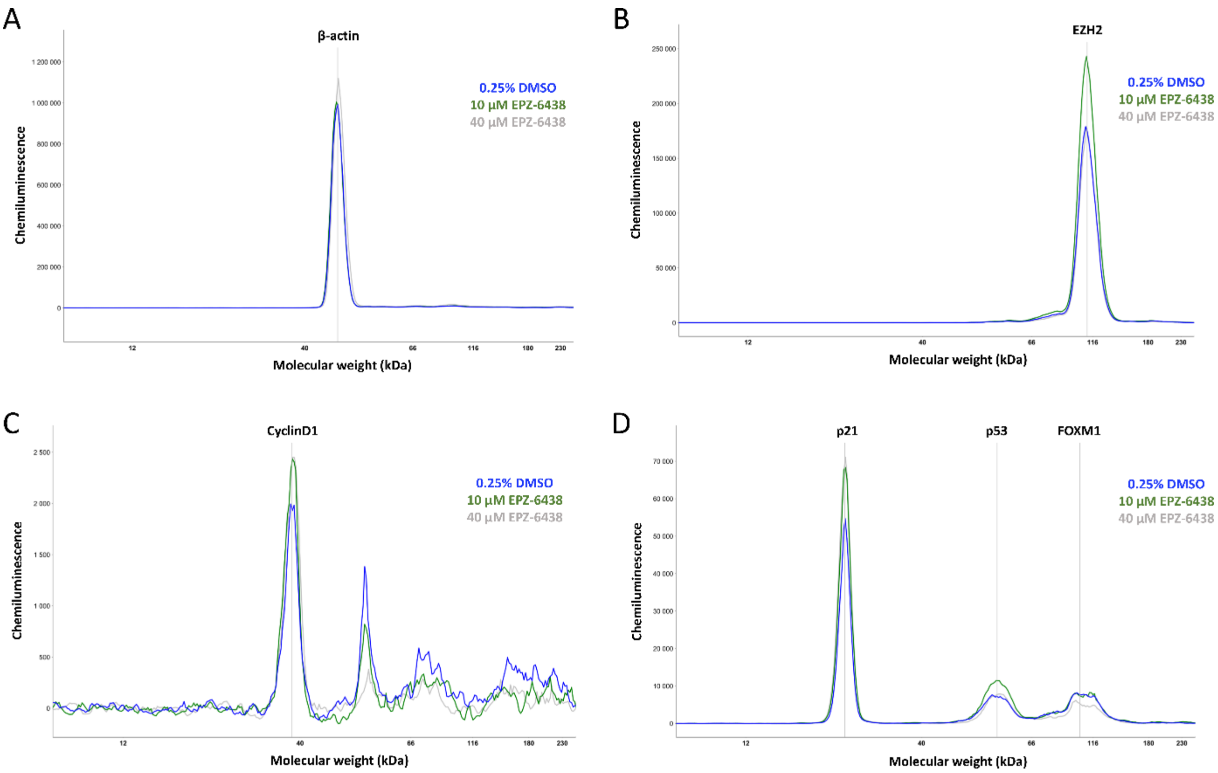

Supplementary Figure S7. Results of one representative Wes^{TM} Simple Capillary Immunoassay analysis. Unadjusted electropherograms demonstrate (**A**) β-actin (loading control), (**B**) EZH2, (**C**) cyclinD1 as well as (**D**) p21, p53 and FOXM1 expression in DMSO (blue), 10 µM (green) and 40 µM (grey) EPZ-6438-treated cells.
